# EGFR activity addiction facilitates anti-ERBB based combination treatment of squamous bladder cancer

**DOI:** 10.1038/s41388-020-01465-y

**Published:** 2020-09-25

**Authors:** Michael Rose, Angela Maurer, Julia Wirtz, Andreas Bleilevens, Tanja Waldmann, Maximilian Wenz, Marie Eyll, Mirja Geelvink, Melanie Gereitzig, Nadine Rüchel, Bernd Denecke, Elke Eltze, Edwin Herrmann, Marieta Toma, David Horst, Tobias Grimm, Stefan Denzinger, Thorsten Ecke, Thomas Alexander Vögeli, Ruth Knuechel, Jochen Maurer, Nadine T. Gaisa

**Affiliations:** 1 Institute of Pathology, RWTH Aachen University, Aachen, Germany; 2 Department of Gynecology, University Clinic RWTH, Aachen, Germany; 3 IZKF Aachen, Medical Faculty of the RWTH Aachen University, Aachen, Germany; 4 Institute of Pathology, Saarbrücken-Rastpfuhl, Saarbrücken, Germany; 5 Department of Urology, University Hospital Münster, Münster, Germany; 6 Institute of Pathology, University Hospital Gustav Carus TU Dresden, Dresden, Germany; 7 Institute of Pathology, Charité – Universitätsmedizin Berlin, Berlin, Germany; 8 Department of Urology, LMU Munich, Munich, Germany; 9 Department of Urology, University of Regensburg, Regensburg, Germany; 10 Department of Urology, Helios Hospital Bad Saarow, Bad Saarow, Germany; 11 Department of Urology, RWTH Aachen University, Aachen, Germany; 12 Present Address: Institute of Pathology, University Hospital Bonn, Bonn, Germany

**Keywords:** Bladder cancer, Growth factor signalling

## Abstract

Recent findings suggested a benefit of anti-EGFR therapy for basal-like muscle-invasive bladder cancer (MIBC). However, the impact on bladder cancer with substantial squamous differentiation (Sq-BLCA) and especially pure squamous cell carcinoma (SCC) remains unknown. Therefore, we comprehensively characterized pure and mixed Sq-BLCA (*n* = 125) on genetic and protein expression level, and performed functional pathway and drug-response analyses with cell line models and isolated primary SCC (p-SCC) cells of the human urinary bladder. We identified abundant EGFR expression in 95% of Sq-BLCA without evidence for activating *EGFR* mutations. Both SCaBER and p-SCC cells were sensitive to EGFR tyrosine kinase inhibitors (TKIs: erlotinib and gefitinib). Combined treatment with anti-EGFR TKIs and varying chemotherapeutics led to a concentration-dependent synergism in SCC cells according to the Chou-Talalay method. In addition, the siRNA knockdown of EGFR impaired SCaBER viability suggesting a putative “Achilles heel” of Sq-BLCA. The observed effects seem Sq-BLCA-specific since non-basal urothelial cancer cells were characterized by poor TKI sensitivity associated with a short-term feedback response potentially attenuating anti-tumor activity. Hence, our findings give further insights into a crucial, Sq-BLCA-specific role of the ERBB signaling pathway proposing improved effectiveness of anti-EGFR based regimens in combination with chemotherapeutics in squamous bladder cancers with wild-type EGFR-overexpression.

## Introduction

Bladder cancer is the 9th common cancer worldwide [1] comprising a wide spectrum of tumors including cancers with squamous differentiation (Sq-BLCA), i.e., urothelial cancers with substantial squamous-differentiation (MIX-SCC) and pure squamous cell carcinoma (SCC) [2]. Sq-BLCA shows shorter overall survival compared with pure urothelial cancer [3]. Accumulating evidence suggests poor response of muscle-invasive bladder cancer (MIBC) with squamous features to standard chemotherapy (MVAC—methotrexate, vinblastine, doxorubicin, and cisplatin or GC—gemcitabine and cisplatin) [4]. In the last years accumulating data have suggested implications of molecular subtypes of bladder cancer for therapeutic options [5–7]. In particular, basal tumors with squamous features may benefit from (neoadjuvant) chemotherapeutic regimens [8]. In 2014, Rebouissou and colleagues suggested the basal-like bladder cancer subgroup to be sensitive for anti-EGFR treatment. Increased activation of the EGFR signaling pathway was shown to correlate with enhanced anti-EGFR drug sensitivity in vitro and in vivo [9].

The ERBB receptor tyrosine kinase (TK) family, consisting of the transmembrane growth factor receptors EGFR (ERBB1), ERBB2 (HER2), ERBB3, and ERBB4, activates numerous downstream pathways including RAS-ERK and PI(3)K-AKT signaling in response to extracellular ligand binding [10], thereby orchestrating intracellular processes like differentiation, migration, and proliferation [11]. In carcinogenesis, aberrant TK activity, triggered by overexpression, point mutations, in-frame deletions and autocrine ligand-receptor simulation [12] drives growth and progression of various tumor types including breast [13] and head and neck [14] cancer. Since trastuzumab, an anti-ERBB2 antibody for the treatment of breast cancer, was approved in 1998, various selective inhibitors (antibodies or small molecules) of ERBB TK have been shown to be effective in different tumor entities either overexpressing EGFR (e.g., head and neck squamous cell carcinoma (HNSCC)) [15] or exhibiting activating *EGFR* mutations (e.g., non-small cell lung cancer (NSCLC)) [16]. In studies of EGFR expression in bladder cancer EGFR overexpression varied strongly between 27 to 74% [17–19**]**, which may be due in part to heterogeneous cohorts and different histopathological and molecular subtypes [20]. Importantly, unselected clinical studies assessing EGFR inhibitors in patients with MIBC failed to demonstrate superior treatment efficacy of combined chemotherapy compared to standard chemotherapy alone [21].

In the present study we gained insights into the usability of EGFR TKI treatment specifically for pure and mixed squamous bladder cancer. Our functional in vitro findings provide evidence that the viability of SCC-derived cells strongly depends on ERBB signaling suggesting anti-EGFR TKI therapy as a valid target, in particular when combined with standard chemotherapy.

## Results

### Genetic alterations and expression of members of the ERBB signaling pathway in urothelial BLCA and Sq-BLCA

**Fig. 1:**
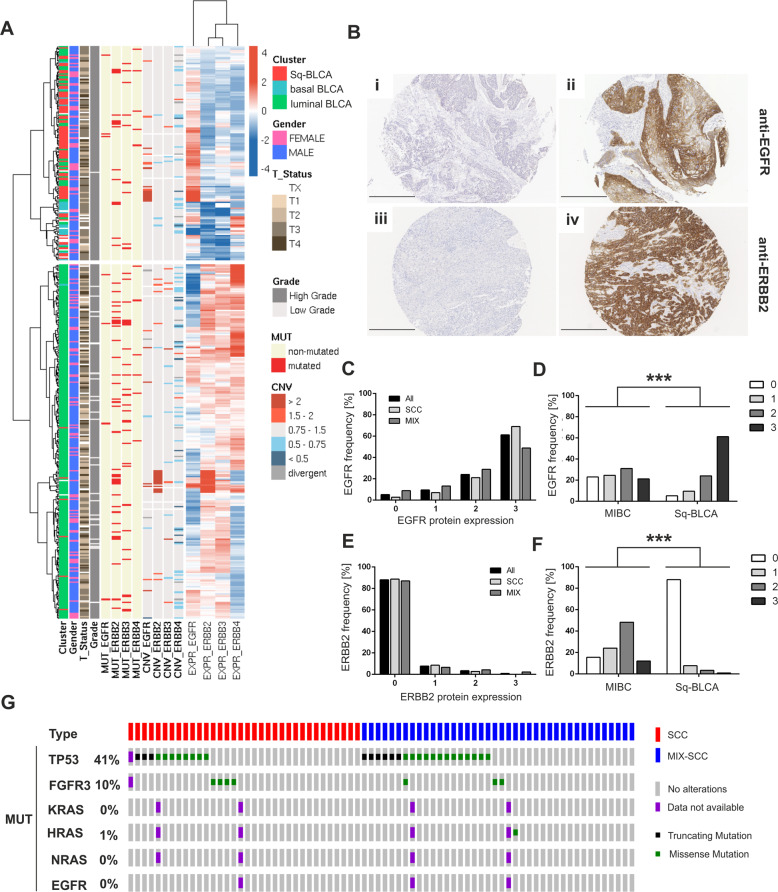
Genetic, transcriptional, and protein alterations of the ERBB pathway in urothelial and squamous bladder cancer. **a** Heatmap of mRNA expression of the ERBB receptor family for the BLCA TCGA data set. Samples are annotated with subtype cluster (red = Sq-BLCA, *n* = 85; blue = basal BLCA, *n* = 21; and green = luminal BLCA, *n* = 280), gender, stage, grade as well as mutational status and CNVs for EGFR and ERBB2–4. **b** Immunohistochemical EGFR (i and ii) and ERBB2/HER2 (iii and iv) protein expression of representative tumor tissues evaluated as intensity 0 and 3, respectively. Black scale bar: 100 µM**. c** Graph shows the frequency of EGFR protein intensity for Sq-BLCA samples (all; *n* = 116) and for subclasses mixed (MIX; *n* = 45) and pure SCC (SCC; *n* = 71). **d** Comparison of immunohistochemical results for EGFR protein expression between Sq-BLCA and urothelial carcinomas (muscle-invasive bladder cancer, MIBC) (Sq-BLCA *n* = 116, MIBC *n* = 63). **e** Graph illustrating the frequency of ERBB2/HER2 protein intensity for all Sq-BLCA (*n* = 117) and for subclasses mixed (MIX; *n* = 46) and pure SCC (SCC; *n* = 71). **f** Comparison of immunohistochemical results for ERBB2 protein expression between squamous-differentiated bladder tumors and MIBC (Sq-BLCA *n* = 117, MIBC *n* = 63). **g** Oncoprint graph highlighting mutations of genes involved in the ERBB signaling pathway (*RAS* genes, *EGFR*). *FGFR3*, and *TP53* mutation analysis served as control (for detailed information on identified mutations see Supplementary Table 2); ***p < 0.001 (Mann–Whitney *U* test).

TCGA bladder cancer data (*n* = 386) were classified into distinct subgroups, i.e., luminal, basal and basal tumors with squamous features [22]. We identified 85 “squamous-like” bladder cancers (Sq-BLCA) with basal characteristics and a squamous-like gene expression profile. Twenty-one bladder cancers showed only basal characteristics (pure basal BLCA). 280/386 bladder cancers were classified as luminal-like/non-basal BLCA. Basal-type cancers with and without SCC features were characterized by abundant *EGFR* mRNA expression (Fig. 1a). Other ERBB-family-receptors (*ERBB2*, *ERBB3*, and *ERBB4*) were barely expressed in basal cancers, whereas non-basal BLCA showed increased mRNA expression of different ERBBs.

Next, EGFR and ERBB2/HER2 protein expression was evaluated in a large cohort of bladder cancers with substantial squamous differentiation comprising MIX-SCC (*n* = 50) and pure SCC (*n* = 75) (Table 1). Urothelial BLCA (MIBC, *n* = 63) without squamous differentiation served as reference (Supplementary Table 1). In 95% (110/116) of Sq-BLCA EGFR protein was expressed (Fig. 1b and c). 61% of these tumors (71/116) showed EGFR overexpression (score 3) (mean score: 2.41 ± 0.87; 95% CI: 2.26–2.57) (Fig. 1c), whereas 21% of MIBC exhibited comparable values (mean score: 1.51 ± 1.07; 95% CI: 1.23–1.78) (Fig. 1d). Conversely, 4% (5/117) of Sq-BLCA expressed ERBB2/HER2 (HER2 DAKO score of 2 or 3) (mean score: 0.17 ± 0.51; 95% CI: 0.08–0.27) (Fig. 1e), whereas 60% of MIBC showed ERBB2/HER2 protein expression (mean score: 1.57 ± 0.90; 95% CI: 1.33–1.81) (Fig. 1f). There was no difference between MIX-SCC and SCC.

**Table 1:** Clinico-pathological data of squamous-differentiated bladder cancers used in this study (Sq-BLCA; *n* = 125).

Variables	SCC (*n* = 75)	MIX-SCC (*n* = 50)
Patient age (years)		
30–49	11	3
50–69	32	18
70–99	29	26
na	3	3
Gender		
Female	35	28
Male	36	19
na	4	3
Tumor stage		
pT1	1	0
pT2	11	6
pT3	43	36
pT4	13	5
pTx	7	3
Tumor grade		
G1	1	0
G2	26	10
G3	44	35
G4	0	2
na	4	3
Nodal status		
N0	44	29
N1	8	6
N2	4	5
na	19	10

*na* not available.

In parallel, genetic EGFR alterations were studied, i.e., *EGFR* amplification, *EGFR* activating mutations, and activating *RAS* mutations (HRAS, KRAS, NRAS) which would convey resistance to EGFR inhibitor treatment. No activating mutations in the *EGFR* gene (0/71) and only a single activating *RAS* mutation (1/69; HRAS p.Q61L) was identified (Fig. 1g and Supplementary Table 2). *EGFR* and *ERBB2* copy number analysis by FISH revealed an amplification of the *EGFR* gene in 8% (9/115) and of *ERBB2* in 0% (0/105). *EGFR* cluster amplifications overlapped with strong EGFR protein expression (7/9) (Supplementary Table 3).

### Efficacy of EGFR TKI and chemotherapeutical treatment on urothelial and SCC-derived cancer cells

**Fig. 2:**
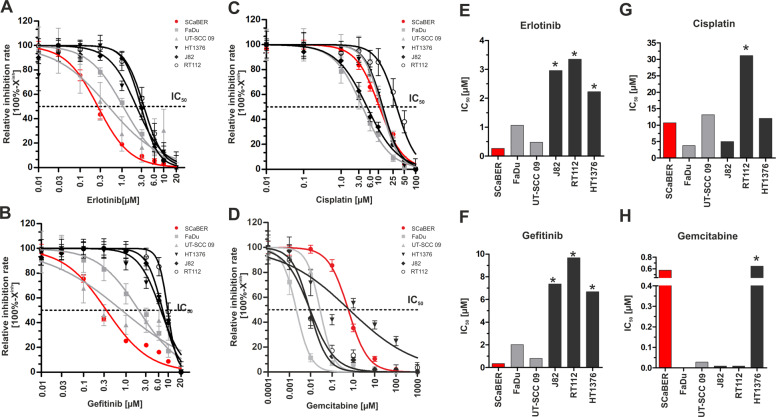
Single drug response analyses applying anti-EGFR TKIs and chemotherapeutical agents on urothelial, squamous bladder, and squamous head and neck cancer cell lines. **a**–**d** Semi-logarithmic plots show drug response curves (relative inhibition rate = 100%−X^inh^) of urothelial bladder cancer cell lines (black lines; RT112, J82, HT1376), a squamous-differentiated bladder cancer cell line (red line; SCaBER) and head and neck cancer cell lines as controls (gray lines; UT-SCC 09 and FaDu) according to erlotinib (**a**), gefitinib (**b**), cisplatin (**c**), and gemcitabine (**d**). Drug response was determined using XTT following 72 h incubation with indicated drug concentrations. **e**–**h** Relative IC_50_ (drug concentration causing 50% inhibition) values are derived from the drug response curve to define the sensitivity of cell lines, respectively. (**e**) erlotinib, (**f**) gefitinib, (**g**) cisplatin and (**h**) gemcitabine. Drug-response curves represent means from at least *n* = 3 independent experiments. *Note: These cell lines did not achieve two assay concentrations at the lower plateau which results in underestimated IC_50_ values.

First-generation tyrosine kinase inhibitors (TKIs), erlotinib, and gefitinib are known to target wild-type EGFR by competing reversibly with adenosine triphosphate (ATP) at the kinase domain [23]. Single drug sensitivity assays were performed (Fig. 2a–d) on SCaBER cells and urothelial cancer cell lines (HT1376, RT112, J82) to calculate relative IC_50_ values for each cell line and drug (Fig. 2e–h). The oropharyngeal cancer cell lines FaDu and UT-SCC 09 served as control groups for pure squamous cancer cells. Expression of ERBB genes (Supplementary Fig. 1) and the status of *EGFR* amplification, *EGFR* or *RAS* activating mutations for used cell lines have been assessed (Supplementary Table 4).

SCaBER was by far the most sensitive cell line tested (erlotinib: IC_50_ = 0.27 µM, gefitinib: IC_50_ = 0.35 µM). FaDu and UT-SCC 09 exhibited high to intermediate sensitivity to both inhibitors (FaDu: erlotinib-IC_50_ = 1.07 µM, gefitinib-IC_50_ = 2.01 µM; UT-SCC 09: erlotinib-IC_50_ = 0.49 µM, gefitinib-IC_50_ = 0.80 µM). The basal bladder cancer cell line HT1376 showed intermediate sensitivity to erlotinib (erlotinib-IC_50_ = 2.23 µM) but not to gefitinib treatment (gefitinib-IC_50_ = 6.68 µM). RT112 and J82 cells were poorly sensitive (erlotinib-IC_50_ > 3 µM; gefitinib-IC_50_ > 7 µM) to both inhibitors. In parallel, we determined the response to gemcitabine and cisplatin as known standard chemotherapeutics for bladder cancer. SCaBER cells were less sensitive to cisplatin (IC_50_: 10.71 µM) or gemcitabine (IC_50_: 0.58 µM) compared to all other cell lines.

### ERBB signaling in response to EGF stimulation, EGFR inhibition, and siRNA-mediated EGFR knockdown in urothelial- and Sq-BLCA cells

**Fig. 3:**
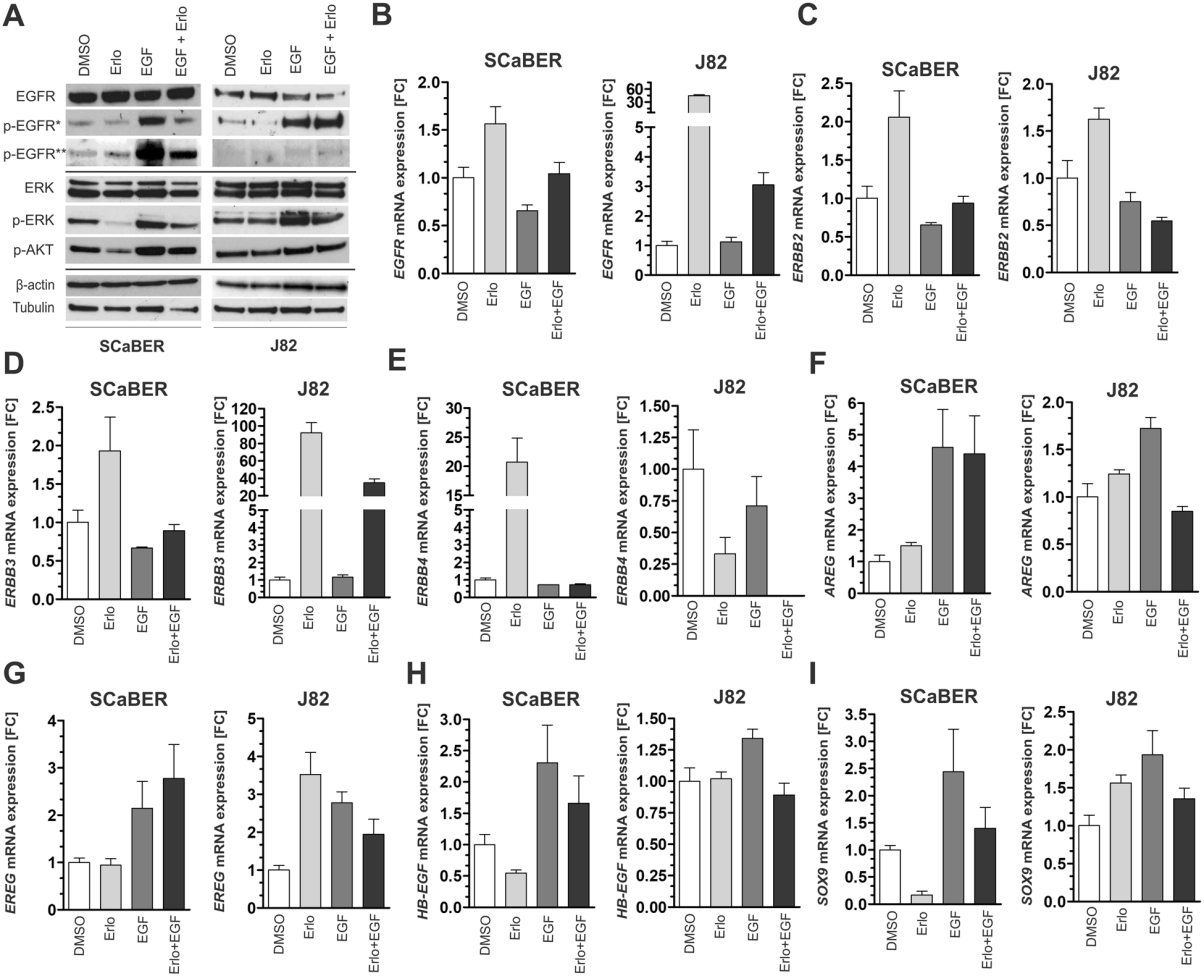
ERBB signaling in SCaBER and J82 bladder cancer cells upon TKI treatment and EGF stimulation. **a** Western blot analyses illustrate activation and inhibition of EGFR/p-EGFR (Tyr1068, Tyr1045), ERK/p-ERK (Thr202,Tyr204), and p-AKT (Ser473) 24 h after EGF and erlotinib treatment. DMSO application was used as untreated control. β-actin (for EGFR) and tubulin (for ERK) served as loading controls. **b**–**i** Relative mRNA expression of ERBB receptors (*EGFR, ERBB2, ERBB3,* and *ERBB4*), ERBB ligands (*AREG, EREG*, and *HB-EGF*) and the EGFR target gene *SOX9* normalized to corresponding DMSO control 24 h after EGF and/or erlotinib treatment for SCaBER and J82 bladder cancer cells. *GAPDH* was used for standardization. FC: fold change. Vertical lines: +standard error of mean (SEM) of triplicates.

SCaBER, J82, and HT1376 cells were treated with EGF (10 ng/ml), erlotinib (determined IC_50_ for each cell line) or combined for 24 h (Fig. 3a). For detailed densitometric evaluation see Supplementary Figs. 2–4. Under basal conditions in SCaBER, 47% of total EGFR protein showed phosphorylation that was associated with 82% activation of the downstream kinase ERK. Upon erlotinib treatment the signaling cascade downstream of EGFR was abrogated by Δp-ERK: 31.8% (Δp-ERK) and Δp-AKT: 29.8% (Δp-AKT). EGF stimulation fostered signaling, i.e. p-EGFR protein up to 125%, p-ERK and p-AKT up to 89.5% and 106%, respectively, while their activation was blocked by 45.8% (Δp-EGFR Tyr1068), 38.2% (Δp-EGFR Tyr1045), 23.1% (Δp-ERK) and 15.9% (Δp-AKT) due to simultaneous erlotinib treatment. ERK activation was also diminished by erlotinib in HT1376 bladder cancer cells (Supplementary Fig. 4), in particular, reduced by erlotinib under stimulatory conditions (Δp-ERK: 51.7%). In urothelial J82 cells only a slight inhibitory effect was observed upon treatment with both erlotinib alone and in combination with EGF (Δp-ERK 8% and 22.6%) while AKT showed even increased phosphorylation (Δp-AKT 17.7% and 8.5%).

A feedback mechanism potentially regulating ERBB receptor and ligand expression was studied (Fig. 3b–i). Twenty-four hours after EGF and/or erlotinib treatment mRNA expression of both ERBB receptors (*EGFR, ERBB2, ERBB3*, and *ERBB4*) and ERBB ligands (amphiregulin (*AREG*), epiregulin (*EREG*), and heparin-binding EGF like growth factor (*HB-EGF*)) [24] was determined. In addition, the transcriptional regulator SRY-related high-mobility group box 9 (*Sox9*) (Fig. 3b–i) was selected as known target gene of the EGFR-ERK axis [25]. Compared to the DMSO control, erlotinib treatment caused a slight upregulation of *ERBB2* (fold change (FC): 2.1) and *ERBB3* (FC: 1.9) mRNA expression, however, re-expression was shown for *ERBB4* mRNA in SCaBER (FC: 20.7) (Fig. 3e) and HT1376 cells (FC: 3.4) (Supplementary Fig. 4). EGFR inhibition by erlotinib further reduced *SOX9* (FC: 0.16) and *HB-EGF* expression (FC: 0.55) while mRNA levels of the low-affinity ligands AREG and EREG were not significantly altered in SCaBER cells. EGF stimulation resulted in an upregulation of *AREG* and *EREG*. As EGF stimulation counteracts the impact of erlotinib upon combined treatment a complete inhibition of the EGFR signaling cascade was not expected (see Fig. 3a) and expression of receptors or ligands were consequently not substantially altered compared to DMSO controls. Interestingly, in non-basal and TKI resistant cancer cells (J82), inhibition of EGFR phosphorylation mediated by erlotinib was associated with increased mRNA expression of *EGFR* (FC: 44.6), *ERBB3* (FC: 92.3) and *EREG* (FC: 3.5). Upregulation of *EGFR* and *ERBB3* was still present upon EGF stimulation in combination with erlotinib (Fig. 3b and d) suggesting a feedback loop with potentially compensatory effects as the target gene *SOX9* was not downregulated by applied TKIs (FC: 1.6) (Fig. 3i).

**Fig. 4:**
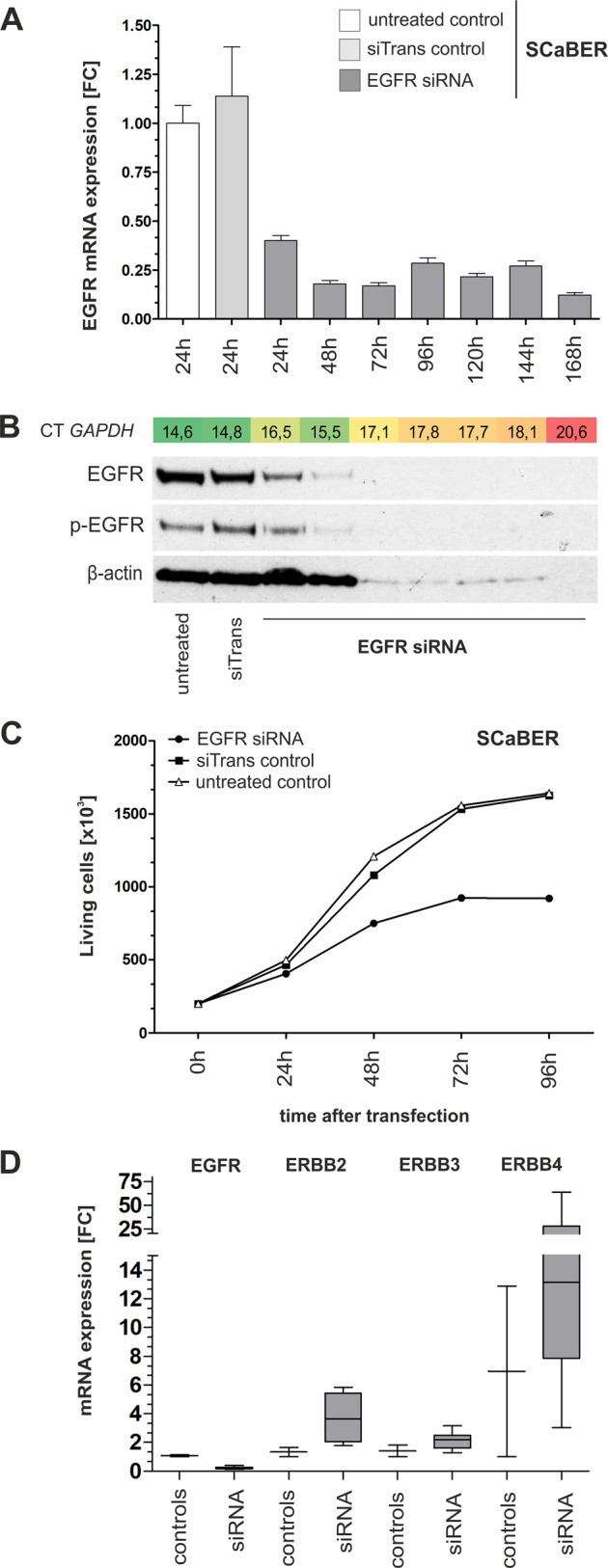
Cell viability and ERBB receptor expression due to siRNA-mediated knockdown of EGFR in SCaBER cells. **a** siRNA-mediated knockdown of EGFR in SCaBER cells is representatively shown for *n* = 3 independent replicates. EGFR knockdown was observed on mRNA level after 48 h (**a**) and on protein level after 72 h (**b**). FC: fold change. Vertical lines: +SEM of triplicates. Seventy-two hours after transfection the number of living cells included in the qPCR and western blot analysis was dramatically reduced—indicated by the loss of the loading control β-actin and a clearly reduced housekeeping gene on mRNA level (CT GAPDH). **c** Cell growth assays confirmed reduced cell viability (living SCaBER cells) due to EGFR knockdown compared with controls. **d** Relative mRNA expression of ERBB receptors (*EGFR, ERBB2, ERBB3*, and *ERBB4*) is shown for SCaBER cells treated with siRNA controls (24 h) and siRNA- knockdown of EGFR (24–168 h for siRNA). Horizontal lines: grouped medians. Boxes: 25–75% quartiles. Vertical lines: range, peak and minimum.

The knockdown of EGFR by siRNA led to impaired cell viability 48 h after siRNA transfection in SCaBER cells (Fig. 4a–c). The number of living cells at different time points after siRNA transfection was reduced by up to 43.3% (Fig. 4c). Basal apoptosis was not affected by EGFR knockdown (data not shown). No impact on cell viability was observed in urothelial J82 cancer cells over 96 h (Supplementary Fig. 5). Expression analyses of *ERBB* receptors revealed an EGFR-knockdown associated upregulation of *ERBB2* and *ERBB4* in SCaBER (Fig. 4d) while J82 cancer cells did not show upregulation of *ERBB* expression at all (Supplementary Fig. 5).

### Combined application of anti-EGFR TKIs and chemotherapeutics enhances drug efficacy in vitro

**Fig. 5:**
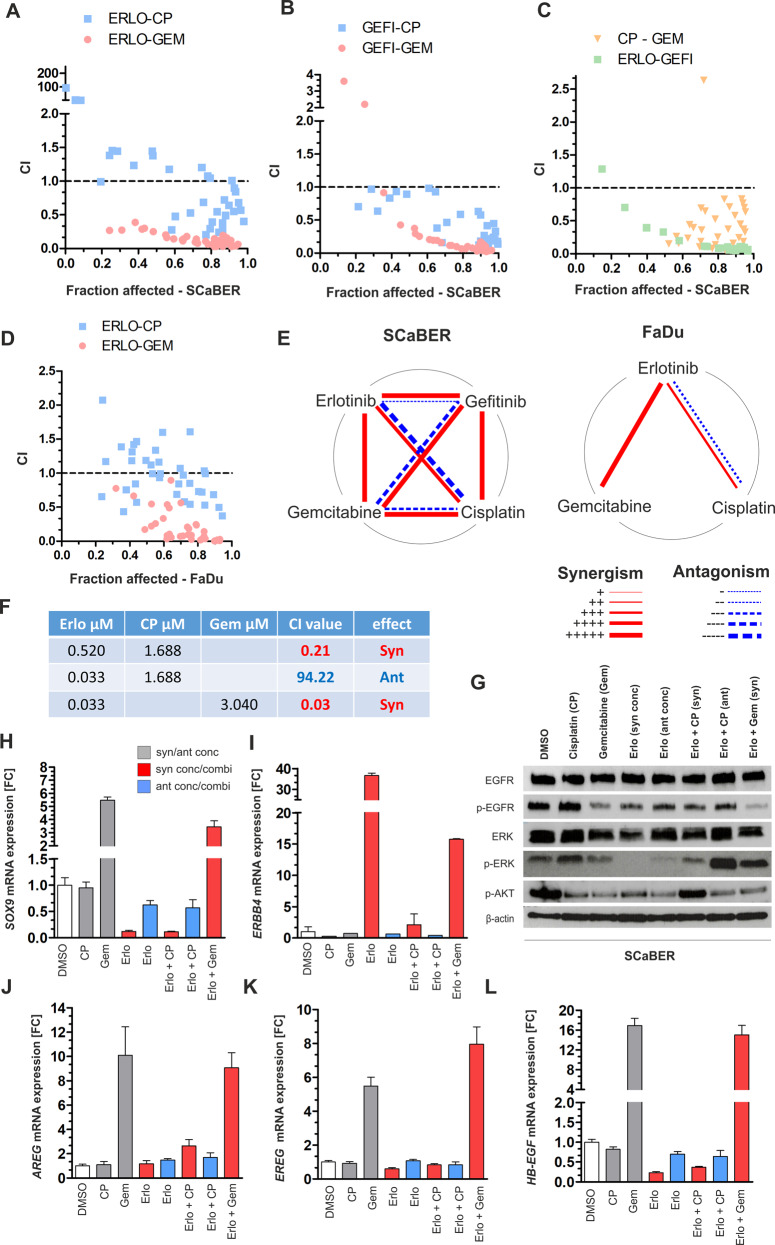
Impact of combined treatment of EGFR TKIs and varying chemotherapeutics on squamous-differentiated bladder cancer cells. Combination Index (CI) was calculated in order to assess the combined effects of drugs (CompuSyn software, v.1.0). **a**–**c** Graphs illustrating CI results for fractions affected by combined application of indicated drugs (ERLO: erlotinib; GEFI: gefitinib; CP: cisplatin; GEM: gemcitabine) on SCaBER cells after 72 h. Drugs were used at concentrations of 4x IC_50_, 2× IC_50_, 1× IC_50_, 0.5× IC_50,_ 0.25× IC_50_, 0.125× IC_50_. CI = 1: additive effect, CI < 1: synergism, CI > 1: antagonism. **d** Fractions affected of FaDu cells by combined application of indicated drugs (ERLO: erlotinib; CP: cisplatin; GEM: gemcitabine). Drugs were used at concentrations of 4× IC_50_, 2× IC_50_, 1× IC_50_, 0.5× IC_50,_ 0.25× IC_50_, 0.125× IC_50_. CI = 1: additive effect, CI < 1: synergism, CI > 1: antagonism. Data represent means from at least *n* = 3 independent experiments. **e** Polygonograms summarizing the effects (synergism/antagonism) according to Chou 2010 [32] of drug combinations for erlotinib, gefinitinib, cisplatin, and gemcitabine on SCaBER and FaDu cells. FaDu served as a technical “squamous” control cell line. **f** Applied drugs and concentrations for EGFR signaling studies. **g** Representative western blot analyses illustrate activation and inhibition of EGFR/p-EGFR (Tyr1068), ERK / p-ERK (Thr202, Tyr204), and p-AKT (Ser473) 24 h after treatment with indicated drug concentrations and combinations. DMSO application was used as an untreated control. β-actin served as loading control. **h**–**l** Relative mRNA expression of the EGFR target gene *SOX9*, the ERBB receptor *ERBB4*, and ERBB ligands (*AREG, EREG*, and *HB-EGF*) normalized to corresponding DMSO control is shown 24 h after treatment of SCaBER cells with indicated drugs. *GAPDH* was used for standardization. FC: fold change. Vertical lines: +SEM of triplicates. Data (western blot and mRNA expression) were confirmed by *n* = 3 independent experiments.

Next, drug-response assays with combinations of erlotinib, gefitinib, and two routinely used chemotherapeutics were performed for SCaBER and FaDu cells (Fig. 5a–d). A combined drug effect was determined by calculating the combination index (CI) as a non-constant combination following the Chou-Talalay method [26] whose results are summarized as polygonograms in Fig. 5e. CI results are shown for SCaBER in Fig. 5a to c according to the fraction affected by combined treatment. Strong synergism was shown for erlotinib equal to or higher than its IC_50_ value which caused CI values ranging between 0.20 and 0.72 in combination with different concentrations of cisplatin (Fig. 5a). CI values in the range of 0.84–94.22 were calculated at lower concentrations of both drugs reflecting antagonistic effects. Combined erlotinib and gemcitabine treatment revealed an unambiguous synergism for all tested combinations (CI range: 0.03–0.39). Combinations of gefitinib and cisplatin showed synergistic effects for almost all applied doses (CI range: 0.077–0.784). A dose-dependent range of CI was calculated while using combined gefitinib-gemcitabine application reflecting either synergism at high concentrations (CI range 0.02–0.15) or both synergism and antagonism at low concentrations (CI range 0.03–3.60) (Fig. 5b). Furthermore, combination of the two chemotherapeutics cisplatin and gemcitabine achieved the expected strong synergism (Fig. 5c) which is in line with previous reports [27]. Combined TKI treatment also improved efficacy with strong synergism for almost all applied dose ranges following a clear dose-dependent effect.

Analyzing the response patterns of the EGFR signaling cascade upon combined treatment of SCaBER, reduced activity for the EGFR-ERK axis was observed only for erlotinib–cisplatin combinations associated with synergistic CI values (Fig. 5f–g). In contrast, combined erlotinib-cisplatin treatment mediating antagonistic effects correlated with increased ERK activation compared to the DMSO control. Cisplatin alone was also associated with p-ERK upregulation. Interestingly, there was a switch from p-ERK to p-AKT activation when comparing combined erlotinib-cisplatin treatment depending on synergistic/antagonistic doses. Overall, gemcitabine treatment (alone and in combination) was associated with reduced EGFR and AKT activation, whereas phosphorylation of the downstream kinase ERK was not clearly blocked.

Considering the transcriptional regulation of EGFR pathway members (Fig. 5h–l and Supplementary Fig. 6), we confirmed the downregulation of *SOX9* only for drug concentrations and combinations mediating synergisms and exhibiting an inactive EGFR-ERK axis, i.e., erlotinib (FC: 0.12) and erlotinib-cisplatin (FC: 0.11). Consistently concentrations/combinations associated with synergistic CI values caused slight or strong re-expression of *ERBB4*. Interestingly, the combined erlotinib-gemcitabine treatment also showed *ERBB4* upregulation (Fig. 5i). However, gemcitabine treatment was associated with the upregulation of all measured ERBB ligands (*AREG, EREG, HB-EGF*) as well as *SOX9* suggesting an interfering impact on the EGFR pathway.

### Proof-of-principle: Impact of anti-EGFR based combined treatment on primary SCC-cells ex vivo

**Fig. 6:**
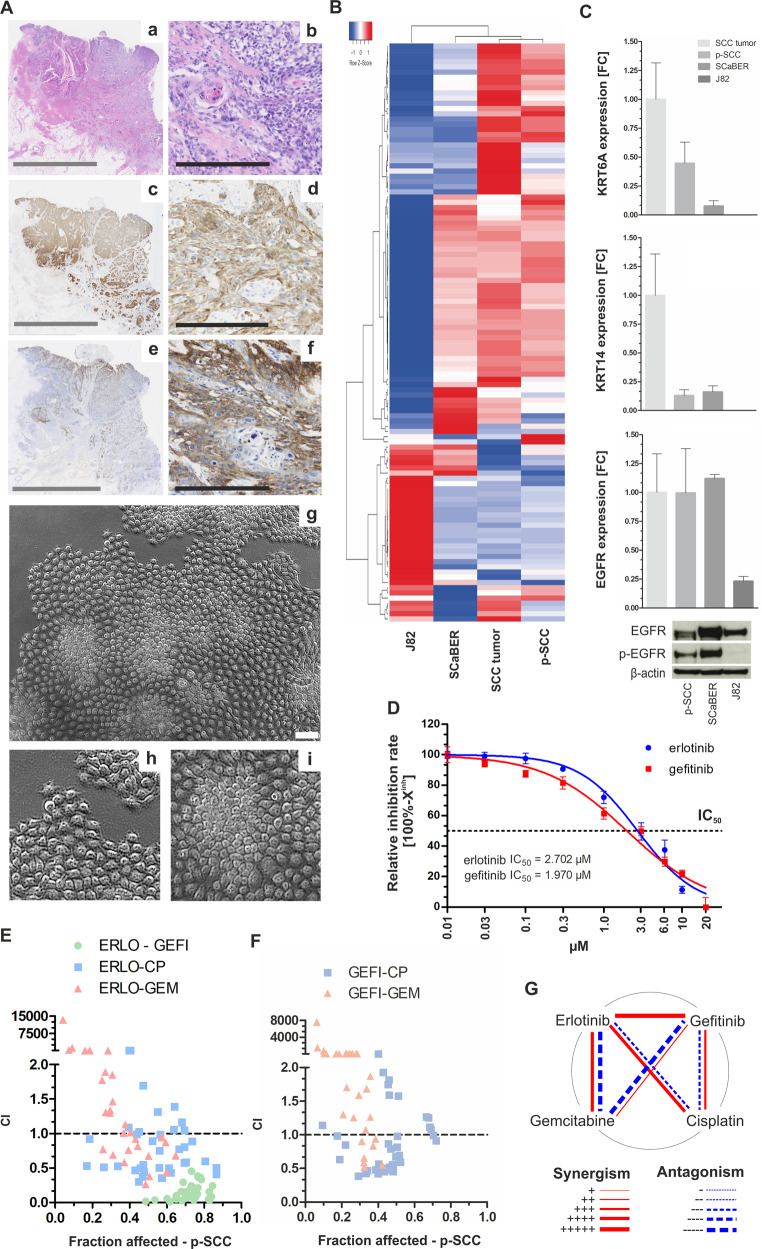
Proof-of-principle of anti-EGFR based treatment of SCC by using primary SCC tumor cells. **a** Histological (**a**–**f**) and morphological (**g**–**i**) characteristics of SCC and derived primary cells. a + b: HE staining of SCC tissue, c + d: Immunohistochemical anti-KRT5/6 staining, e + f: Immunohistochemical anti-EGFR staining, **g**–**i** Phase-contrast images of isolated SCC cells. Gray scale bar: 10 mm; black scale bar: 250 µm; white scale bar: 100 µm. **b** Heatmap of the 111 strongest regulated genes including *KRT6A, KRT14*, and *TP63* illustrating a tight correlation between SCC and derived tumor cells (for detailed gene list see Supplementary Table 5). **c** Confirmation of *KRT6A/14* and *EGFR* mRNA expression in p-SCC normalized to corresponding tumor tissue which is comparable with that of SCaBER cells. J82 served as non-basal control. *GAPDH* was used for standardization. FC: fold change. Vertical lines: + SEM of triplicates. **d** Single drug response analysis applying anti-EGFR TKIs after 72 h incubation. Drug-response curves showing relative inhibition rate (inhibition rate = 100%−X^inh^) of p-SCC cells due to erlotinib and gefitinib treatment. **e**–**f** Graph illustrating CI results for fractions affected by combined application of indicated drugs (ERLO: erlotinib; GEFI: gefitinib; CP: cisplatin; GEM: gemcitabine) on p-SCC cells. Drugs were used at concentrations of 4× IC_50_, 2× IC_50_, 1× IC_50_, 0.5× IC_50,_ 0.25× IC_50_, 0.125× IC_50_. CI = 1: additive effect, CI < 1: synergism, CI > 1: antagonism. Data represent means from *n* = 2 independent experiments. **g** Polygonograms summarize the effects (synergism/antagonism) according to Chou [37] of drug combinations for erlotinib, gefitinib, cisplatin and gemcitabine on p-SCC cells.

Primary cells (p-SCC) derived from pure SCC tissue were established as a cell culture ex vivo model. The tissue of origin showed pure squamous differentiation (Fig. 6a, a + b) with abundant KRT5/6 staining (Fig. 6a, c + d). Strong EGFR expression (score 3) was observed in 20% of SCC cells with an overall heterogeneous staining pattern (e + f). Derived p-SCC cells were cultivated under low oxygen conditions (3% O_2_). The cells displayed an epithelial, cobblestone-like morphology (Fig. 6a, g–i). p-SCC cells were characterized and compared with the original tumor tissue by transcriptomic analysis. SCaBER and J82 cells served as squamous and urothelial-like controls, respectively. In Fig. 6b, the 111 most up- and downregulated genes (FC ≥ 500) are shown in a heatmap (for detailed gene list see Supplementary Table 5). We observed a close correlation between the gene expression pattern of p-SCC cells and the tissue of origin. Most of the overexpressed or weakly expressed genes could be confirmed in SCaBER cells while the urothelial cell line J82 showed a completely different gene expression pattern. mRNA expression of the two basal-type markers *KRT6A* and *KRT14* as well as *EGFR* confirmed comparable levels between the original tumor tissue and p-SCC (Fig. 6c). Total EGFR protein was slightly lower expressed in p-SCC cells than in SCaBER, but a high level of activated EGFR was confirmed for p-SCC cells.

Performing single (Fig. 6d) and combined drug treatment (Fig. 6e and f) the pool of heterogeneous p-SCC cells showed intermediate sensitivity to both TKIs ranging between 1.97 µM (gefitinib-IC_50_) and 2.70 µM (erlotinib-IC_50_) (Fig. 6d) associated with strong inhibition of p-ERK and p-AKT activation as well as *SOX9* expression (FC: 0.17) (Supplementary Fig. 7A and B). Interestingly, ERBBs were not differentially expressed, however, *AREG*, *EREG* and *HB-EGF* were highly downregulated upon TKI mediated EGFR inhibition (Supplementary Fig. 7B). Combinations of erlotinib (Fig. 6e) and gefitinib (Fig. 6f) with cisplatin and gemcitabine resulted in dose-dependent synergistic and antagonistic effects (Fig. 6g). Combined TKI application caused strong synergism that led to effective inhibition of cell viability (>80% fractions affected) supporting a strong dependency of SCC-derived cells on EGFR signaling.

## Discussion

Aberrant activation of ERBB signaling pathways has emerged as an effective therapeutic target in the field of precision medicine [23]. EGFR inhibition by monoclonal antibodies or small molecule TKIs has been approved for the treatment of tumor entities like RAS wild-type colorectal cancers [28], HNSCC [29], and EGFR-mutated NSCLC [16, 30**]**. In the present study, we provide a rationale for combining EGFR inhibitors with standard chemotherapy as treatment strategy for pure and mixed squamous bladder cancers, characterized by a strong dependency on wild-type EGFR signaling. Functionally, we confirmed a central role of wild-type EGFR in squamous-differentiated bladder cancer cells, as they are vulnerable to perturbances of the ERBB signaling pathway in vitro. SCaBER cells, lacking activating mutations or amplifications of the *EGFR* gene, were highly sensitive to treatment with both anti-EGFR TKIs erlotinib and gefitinib. The corresponding IC_50_ value is very close to those reported for EGFR-mutated NSCLC cell lines (example for gefitinib sensitivity: PC-9 (del 746–750) IC_50_ = 0.0235 µM) [30]. An intermediate sensitivity to anti-EGFR TKIs was confirmed in primary SCC cells whose IC_50_ range was comparable to that of oropharyngeal squamous cancer cell lines. Bearing in mind that EGFR is a validated target in HNSCC [29], our in vitro data suggest promising efficacy of anti-EGFR TKIs in Sq-BLCA of the urinary bladder as well. This hypothesis fits to our molecular and functional findings of the ERBB pathway. In SCaBER and p-SCC cells, the activity of the EGF receptor and the downstream kinases ERK/AKT were effectively blocked upon TKI treatment. A feedback loop mechanism upregulating ERBB4 receptors was observed in SCaBER, but feedbacks reinforcing EGFR signaling were not shown, i.e., the target gene SOX9 [25] remained suppressed while EGFR ligands such as HB-EGF were downregulated upon inhibition. A functional knockdown of EGFR by siRNA confirmed strong dependency of SCaBER cells on the ERBB pathway as EGFR loss was associated with reduced cell viability. Hence, squamous-differentiated bladder cancer appears to be oncogenically addicted to EGFR activity and consequently sensitive to both EGFR inhibition and knockdown without any short-term escape mechanisms, thereby suggesting a putative “Achilles heel”.

In contrast, invasive urothelial cancer cells were characterized by poorer sensitivity to treatment with anti-EGFR TKIs (IC_50_ > 3 µM). EGFR inhibition did not result in a significant inactivation of ERK or AKT in J82 cells. A single activating mutation in *ERBB2* (R678Q) may contribute to reduced sensitivity against TKIs by heterodimerization with EGFR. Beyond that, we did not find genetic alterations in EGFR or downstream effectors in J82 cells, which could further mediate an anti-EGFR drug resistance. Interestingly, we observed a short-term feedback response upon erlotinib treatment in J82 cells, i.e., *EGFR*, *ERBB3*, and *EREG* were upregulated. Mutual compensation by other members of the ERBB family has been described as a bypass mechanism to evade ERBB-TKI inhibition, thus the observed feedback may indeed limit the sustained inhibition of the ERBB pathway in J82. In particular trans-phosphorylation of ERBB3 mediated by compensatory pathways like the PI(3)K/AKT signaling cascade has been proposed to bypass the impact of ERBB TKIs [31, 32]. In addition, EREG has been shown to induce weaker EGFR dimers but causing sustained EGFR signaling [33]. As a clinical consequence, ERBB pathway inhibition may have limited overall efficacy in non-squamous urothelial tumor cells relieving the pathway repression which may explain why unselected clinical trials failed to demonstrate the clinical significance of EGFR inhibition in bladder cancer so far [34, 35]. This agrees with Eriksson and colleagues who concluded that the application of EGFR/HER2 inhibitors did not adequately consider the molecular heterogeneity of bladder cancers in clinical trials [36].

By combining EGFR inhibitors and cytotoxic chemotherapeutics, we further revealed strong synergistic effects in SCaBER cells. Interestingly, combinations of different TKIs, i.e., gefitinib-erlotinib, also improved drug efficiency in both SCaBER and p-SCC. As synergisms are thought to be basically a physiochemical mass-action law issue of the drug-receptor interaction, i.e., any reaction is proportional to the concentrations of the reactants [37, 38], synergistic effects may hint at slightly different affinities of both TKIs to EGFR. Beyond that, lack of specificity is assumed for diverse TKIs including erlotinib and gefitinib [39], suggesting putative further targets of strong homology such as ERBB2/HER2 which might be especially noticeable in the pool of p-SCC cells derived from a tumor with heterogeneous EGFR expression. Non-EGFR specific effects might be also responsible for *EGFR* and *ERBB3* upregulation upon erlotinib treatment in J82 cells as we could not confirm similar responses after a functional EGFR knockdown.

Nevertheless, it should be noted that not every combination is necessarily useful. Combined treatment also caused a concentration-dependent antagonism albeit to a lesser extent. Although the mass-action law seems critical, drug potency in pharmacological models is more complex [40] and antagonisms of combined TKI-chemotherapeutic treatment could be discussed in a mechanistically context as well. It has been shown that the application of gefitinib and cisplatin displayed a dose-dependent antagonism in EGFR wild-type and EGFR mutant NSCLC cell lines, revealing an interference of cisplatin cell entry [30] at a concentration range of gefitinib between 0.001–0.3 µM. Tsai et al. concluded that this antagonism might partly explain why randomized trials including standard chemotherapeutics to NSCLC failed to show benefits for this combined regimen [41]. It has been further proven that both the order and the timing of the application of an EGFR inhibitor and a chemotherapeutic agent could be important to achieve clinical benefits for the patients. Cisplatin followed by afatinib exposure caused more cytotoxic effects than the reverse order or simultaneous application [42]. Considering the order of treatment, mechanisms impairing the efficacies of EGFR inhibitors and cisplatin have been reported in both directions [43]. Ahsan et al. have demonstrated antagonistic effects due to an inhibitory impact of EGFR treatment on cisplatin-induced EGFR phosphorylation [44]. In addition, Benhar et al. demonstrated ERK activation induced by cisplatin [45] which fits to the here observed signaling response patterns. It has been further shown that gemcitabine induces ligands of the EGFR pathway such as AREG [46] which was similar to our findings associated with activated EGFR signaling. Thus, attenuating a general gemcitabine-driven activation of the EGFR pathway by simultaneous application of TKIs may support our observed synergistic impact of combined erlotinib-gemcitabine treatment. In light of such interferences with the EGFR pathway clinical usability of TKI-chemotherapy combinations remains questionable from mechanistically aspects as a clear EGFR pathway inhibition was not detectable. However, combined erlotinib-cisplatin treatment reached EGFR signaling inhibition results equal to those of an individual use of erlotinib (without evidence of compensatory effects), but with an additional cytotoxic impact of cisplatin. Since a combination of targeted therapeutics with common treatment strategies is known to improve clinical outcomes of NSCLC patients [47, 48] this option may be an example of how to translate such in vitro data into a clinical study as previously demonstrated for HNSCC [49].

In summary, our study reveals that Sq-BLCA is highly sensitive to inhibition of the wild-type EGFR signaling pathway lacking known intrinsic mechanisms of ERBB-family TKI resistance. Our in vitro data give further evidence that Sq-BLCA patients may benefit from combined treatment with anti-EGFR TKIs and chemotherapeutics, in particular by dual targeting of the EGFR signaling pathway from different sites as previously assessed in a clinical trial [50]. The order and timing of the combinatorial treatment strategy should be considered for future study designs, and histological and molecular testing of bladder cancer prior to treatment might be the key to improve therapeutic management for (Sq-BLCA) patients.

## Materials and methods

### Patient samples

A non-schistosomal squamous bladder cancer cohort (*n* = 75 different pure SCCs, *n* = 50 different urothelial carcinomas with substantial partial (>50%) squamous differentiation) of formalin-fixed paraffin-embedded (FFPE) surgical specimens from collaborating Institutes of Pathology in Germany and the German Study Group of Bladder Cancers (DFBK e.V.) was used (see Table 1). Tissue microarray construction (TMA) has been described previously [51]. For the comparison of EGFR and ERBB2/HER2 protein expression additional TMAs of a MIBC cohort without squamous differentiation (*n* = 63, pure urothelial MIBC) were used (Supplementary Table 1). Due to the limited availability of material, experimental or clinical data, and case numbers vary for different methods as indicated. Clinical data were obtained by the records of the Departments of Urology and the local ethics committee approved the retrospective, pseudonymized study of archival tissues (RWTH EK 009/12). Tumor tissue of an individual diagnosed with a pure SCC was obtained from the RWTH centralized biomaterial bank (RWTH cBMB) for SCC-tumor cell isolation. The patient gave written consent and experiments were in accordance with the regulations of the biomaterial bank and the Institutional Review Board (IRB)-approved protocols of the Medical Faculty (RWTH EK 206/09, study number 199).

### SCC-tumor cell isolation method and cell culture

Isolation and culturing of primary cells (p-SCC) were performed as described previously for BCSCs [52]. For details of primary cell culture and used cell lines see Supplementary Information. If not otherwise stated all further experiments with the human cells were independently performed at least three times.

### Anti-EGFR and anti-ERBB2/HER2 immunohistochemistry

Immunohistochemical staining of 3 µm TMA sections with diagnostically approved anti-EGFR (Clone E30, monoclonal mouse, M7239, DAKO, Hamburg, Germany, 1:10) or anti-ERBB2/HER2 antibodies (c-erbB-2, polyclonal rabbit, A0485, DAKO, 1:300) was performed on an autostainer 360 (Thermo Fisher Scientific, Waltham, USA) as previously specified [53]. For modifications see Supplementary Information.

### DNA extraction and Sanger sequencing

DNA extraction of FFPE tissue samples (*n* = 69 samples) was performed using QIAamp DNA Mini Kit (Qiagen, Hilden, Germany) according to the manufacturer’s instructions. PCR-amplification and Sanger sequencing was done as previously described [54]. For integrative visualization of sequencing data the cBio Cancer Genomics Portal [55] based OncoPrint tool was used [56]. For detailed gene, exon, and primer information see Supplementary Information and Supplementary Table 6.

### Fluorescence in situ hybridization (FISH)

FISH was performed as reported [54] with slight modifications stated in the Supplementary Information.

### RNA extraction and reverse transcription PCR

Total RNA was extracted from cultured cells using TRIzol^TM^ reagent (Thermo Fisher Scientific) or using the Nucleospin RNA Plus Kit (Macherey-Nagel, 740984.50) according to the manufacturer’s instructions as indicated. 1 µg of RNA was used for cDNA reverse transcription by Promega Kit A3500 according to the manufacturer’s instructions (Promega, Mannheim, Germany).

### Semi-quantitative real-time PCR

cDNAs were amplified by real-time PCR using SYBR-Green PCR mix (Bio-Rad Laboratories, Munich, Germany) in an iCycler IQ5 (Bio-Rad Laboratories) as previously described [57]. All primer sequences are listed in Supplementary Table 7. Gene expression was quantified by using the comparative 2^−ΔΔCT^ method calculating relative expression values and *GAPDH* was used for standardization. If the comparison to a reference sample was not applicable, the gene expression was calculated as % expression of the measured *GAPDH* expression (2^−ΔCT^) according to Schmittger and Livak [58].

### Western blot

Western blot analysis was performed as recently described [57] with slight modifications (see Supplementary Information).

### Single and combined drug response assays and pathway analyses

Dose response curves were performed applying the tyrosine-kinase inhibitors erlotinib (LC Laboratories, Woburn, MA) and gefitinib (Selleckchem, München, Germany) and the chemotherapeutics gemcitabine and cisplatin (obtained ready to-use from the in-house pharmacy of the RWTH Aachen University Hospital). Cell viability was determined by adding XTT (Roche Diagnostics, Penzberg, Germany) according to the manufacturer’s instructions. For details see Supplementary information.

### ERBB pathway stimulation and inhibition

For stimulation with recombinant EGF (10 ng/ml) (GIBCO, Thermo Scientific, MA, USA), and/or erlotinib inhibition (conc. as indicated) up to 24 h, SCaBER, J82, and HT1376 cells were cultured in serum-free media, containing human transferrin and 1% DMSO (Sigma-Aldrich). For p-SCC cells standard conditions (see Supplementary Information) were used. Cellular proteins were extracted in RIPA lysis buffer containing phosphatase inhibitors and quantified using the Pierce^TM^ BCA protein assay (Thermo Scientific, MA, USA). RNA was extracted using the Nucleospin RNA Plus Kit.

### RNA interference of EGFR

Cells were transfected with siTran 1.0 siRNA transfection reagent (Origene, Cat. No. TT300002) applying a siRNA directed against EGFR (Origene) according to the manufacturer’s instructions. For details see Supplementary Information.

### Cell growth assay

SCaBER cells were seeded 48 h after transfection into 6-well plates (2 × 10^4^ cells/well). Twenty-four hours later cells were retreated with siRNA. Cell numbers were determined every 24 h, using Casy®-1 cell counter (OLS Bio, Bremen, Germany).

### Apoptosis assay

The Apo-One® Homogeneous Caspase-3/7 Assay (Promega) was used to detect the activity of effector caspases 3 and 7 as previously described [57].

### Microarray analysis

Transcriptomic profiling was performed by the IZKF (Interdisciplinary Centre for Clinical Research Aachen) Chip-Facility using the Clariom D gene array (Affymetrix, Santa Clara, CA). For details see Supplementary Information. The microarray data were uploaded to the National Center for Biotechnology Information Gene Expression Omnibus (GSE146975; reviewer access: kxqbicmorfejnwv).

### TCGA data acquisition

Public BLCA data sets from the Cancer Genome Atlas (TCGA) [59] network were classified and analyzed as described [22].

### Statistics

Statistical analyses were performed using SPSS 25.0 (SPSS, Chicago, IL, USA) and GraphPad Prism 5.0. Differences were considered statistically significant if the two-sided *p*-values were equal or below 5% (≤0.05). The non-parametric Mann–Whitney U-test was used to compare two groups. Results of single and combination drug assays were used to calculate the Combination Index (CI) with Compusyn (version 1.0) [26, 37**]**.

## Supplementary information

Supplementary Information: Detailed description of methods.

Supplementary Figure 1: ERBB receptor expression in urothelial, squamous bladder and head and neck cancer cell lines.

Supplementary Figure 2: Densitometric evaluation of ERBB pathway activation and inhibition in SCaBER cancer cells.

Supplementary Figure 3: Densitometric evaluation of ERBB pathway activation and inhibition in J82 cells.

Supplementary Figure 4: ERBB signaling and receptor expression in HT1376 bladder cancer cells upon TKI treatment and EGF stimulation.

Supplementary Figure 5: ERBB receptor expression after siRNA mediated knockdown of EGFR in J82 cells.

Supplementary Figure 6: ERBB receptor expression (EGFR, ERBB2, ERBB3) upon combined treatment in SCaBER cells

Supplementary Figure 7: EGFR signaling in pSCC cells upon TKI treatment and EGF stimulation.

Supplementary Table 1: Clinico-pathological data of urothelial, muscle-invasive bladder cancers without squamous characteristics used in this study.

Supplementary Table 2: Detailed information on identified mutations in Sq-BLCA (SCC n=34, MIX n=40).

Supplementary Table 3: Detailed information on amplification of EGFR and HER2/ERBB2 in Sq-BLCA

Supplementary Table 4: Molecular characteristics of utilized cell lines.

Supplementary Table 5: p-SCC associated gene signature.

Supplementary Table 6: Primer sequences for Sanger sequencing of FFPE Material.

Supplementary Table 7: PCR primer sequences for ERBB receptor, ligand and target gene expression analysis (intron spanning).
