# Supplementary material for: EGFR activity addiction facilitates anti-ERBB based combination treatment of squamous bladder cancer
Source: Oncogene. 2020 Sep 25;39(44):6856–70. doi: 10.1038/s41388-020-01465-y (PMC7605436; doi:10.1038/s41388-020-01465-y)
Supplement: Supplementary file 1 — Supplementary Information: Detailed description of methods. [file 41388_2020_1465_MOESM1_ESM.docx]

**Supplemental information:**

**Cell culture**

p-SCC cells were cultured in mammary stem cell (MSC) medium [52] as spheres in a 3D 50% Matrigel (Corning, 35423) environment or expanded in 2D on culture plates coated with 2% Matrigel, as described previously for BCSCs [52]. Cells were grown at 37°C under low oxygen conditions (3% O_2_, 5%CO_2_, 92%N_2_). MSC medium was renewed every 3 days and cells were split using Accutase (Sigma-Aldrich, A6964) once a week. The medium is composed of mammary epithelial basal medium (MEBM, Lonza, CC-3151), supplemented with 1 × B27 (Gibco, 17504-044), 1 × amphotericin B (Gibco, 15290-026), and 1 × penicillin–streptomycin (Gibco, 15140-122). Furthermore, epidermal growth factor (20 ng/mL, PeproTech, AF-100-15), heparin (4 μg/mL, Sigma-Aldrich, H3393-25KU), fibroblast growth factor (20 ng/mL, PeproTech, AF-100-18B), gentamicin (35 μg/mL, Gibco, 15750-045), and rho kinase inhibitor (500 nmol/L, Calbiochem, 555552) were added.

Urothelial cancer cell lines (J82, HT1376) were originally obtained from the American Type Culture Collection (ATCC, Manassas, VA). SCaBER, a basal bladder cancer cell line with squamous features, was kindly provided by Prof. Wolfgang Schulz (Düsseldorf University Hospital, Germany). RT112 was obtained by Dr. Alexander Buchner (LMU München, Germany). Two oropharyngeal squamous cell carcinoma cell lines (UT-SCC 09 and FaDu), included as controls, were a gift from Dr. Kathrin Scheckenbach (Düsseldorf University Hospital, Germany). All cell lines successfully underwent an identity check (Multiplexion GmbH, Immenstadt, Germany) prior to the experiments and were regularly tested for mycoplasma infection using the PCR-based Venor^®^ GeM Mycoplasma Detection Kit (Minerva Biolabs, Berlin, Germany).

**Anti-EGFR and anti-ERBB2/HER2 immunohistochemistry**

Immunohistochemical staining of 1-3 µm TMA sections with diagnostically approved anti-EGFR (Clone E30, monoclonal mouse, M7239, DAKO, Hamburg, Germany, 1:10) or anti-ERBB2/HER2 antibody (c-erbB-2, polyclonal rabbit, A0485, DAKO, 1:300) was performed on an autostainer 360 (Thermo Fisher Scientific, Waltham, USA) as previously specified. ErbB2/HER2 staining heat induced antigen retrieval at pH 6.0 (PT Link, DAKO) and for EGFR staining pretreatment with proteinase K was used. After incubation with primary antibodies, the DAKO EnVision^M^FLEX system (mouse or rabbit linker polymere and horseradish peroxidase-conjugated polymer) for detection was applied. Reactions were visualized with DAKO Liquid DAB Substrate Chromogen System and haematoxylin counterstain. Staining were evaluated by an experienced uropathologist (NTG) according to the semiquantitative EGFR DAKO score (0 = negative, 1 = weak, 2 = moderate, 3 = strong positive/overexpressed) or the HER2 DAKO score (0-1 = negative, 2 = moderate positive, 3 = strong positive/overexpressed) respectively.

**DNA extraction and Sanger sequencing**

DNA extraction of FFPE tissue samples (n=69 samples) was performed using QIAamp DNA Mini Kit (Qiagen, Hilden, Germany) according to the manufacturer’s instructions. PCR-amplification of exons 18, 19, 20 and 21 of the *EGFR* gene, exon 2, 3 and 4 of *RAS* genes (HRAS, NRAS, KRAS), exon 5-9 of the *TP53* gene, and exon 7, 10 and 15 of the *FGFR3* gene were performed using standard protocols. Primer sequences, annealing temperatures and PCR product sizes are listed in supplementary material (Supplementary Table 1). Sanger sequencing (ABI PRISM 3500 Genetic Analyzer (Applied Biosystems, Weiterstadt, Germany)) was applied to sequence both DNA strands using the Big dye Terminator v1.1 Cycle Sequencing Kit (Applied Biosystems) according to the manufacturer’s instructions including corresponding primers.

**Fluorescence in situ hybridization (FISH)**

Hybridization of *ZytoLight* Dual Color Probe SPEC EGFR*/CEN* 7 and *ZytoLight* Dual Color Probe SPEC HER2*/CEN* 17 (Zytovision, Bremerhaven, Germany) onto 3 µm TMA sections was performed according to the manufacturer’s protocols. Slides were reviewed on a Zeiss Axiovert 135 fluorescence microscope (Carl Zeiss, Oberkochen, Germany), and Diskus Software (Buero Hilgers, Königswinter, Germany) was used to capture images from different channels/filters (AHF ZyGreen F36-720, AHF ZyOrange F36-740, AHF DAPI, AHF F56-700). The numbers of *EGFR* or *HER2* and centromere signals was determined in nuclei of 60 tumor cells and the gene/centromere ratio was calculated.

**Western Blot**

Protein lysates were prepared under reducing (25 mM DTT) conditions in RIPA + 2x Nupage LDS (1:1) lysis buffer, separated in 4-12% Bis-Tris gels (Invitrogen Life Technologies, Darmstadt, Germany) using MOPS-SDS running buffer and electroblotted onto nitrocellulose membranes (0.45 µm). After blocking in Tris-buffered saline containing 0.01% Tween-20 (TBS-T) and 5% non-fat dry milk (Roth, Karlsruhe, Germany), blots were probed with primary antibodies (ab) EGFR ((D38B1) ab #4267; 1:1000, Cell Signaling Technology, Frankfurt, Germany), p-EGFR ((D7A5) ab #3777: Tyr1068, 1:500 and ab #2237: Tyr1045 1:500, Cell Signaling Technology), ERK1,2 ((137F5) ab #4695 1:1000, Cell Signaling Technology), p-ERK1,2 ((197G2) ab #4377; Thr202,Tyr204, 1:500, Cell Signaling Technology) or p-AKT ((D9E) ab #4060; 1:500 Cell Signaling Technology) in blocking solution buffer, washed and incubated with goat anti-rabbit 1:1000 (Cell Signaling Technology) secondary peroxidase-conjugated antibodies. Antibody detection was accomplished with Pierce ECL Western Blotting Substrate (Thermo Scientific, Rockford, IL). Equal protein loading was monitored by using either β-actin (ab A5441; 1:1000, Sigma-Aldrich) or β-tubulin ((9F3) ab #5346; 1:1000 Cell signaling Technology) specific antibody.

**Single and combined drug response assays and pathway analyses**

Cells were seeded in DMEM supplemented with 0.5% fetal calf serum and 40 µg/ml transferrin (Sigma-Aldrich, Deisenhofen, Germany). After 72 h treatment of cells with various doses of cisplatin (0.01 µM – 100 µM in H_2_O), gemcitabine (0.0001 µM – 1,000 µM in H_2_O), erlotinib (0.01 µM – 20 µM in 0.1% DMSO) and gefitinib (0.01 µM – 20 µM in 0.1% DMSO), cell viability was determined by adding XTT (Roche Diagnostics, Penzberg, Germany) according to the manufacturer’s instructions. Optical density was measured with an ELISA-Reader Infinite M200 Tecan (Bio-Rad). Each assay was independently performed at least three times. Logarithmic transformation, normalization (defining smallest value = 0% and largest value = 100%) and non-linear regression of raw data was performed using GraphPad prism 6 software (GraphPad Software Inc., La Jolla, CA). The relative inhibition rate (100%-X^inh^) and the IC_50_ (drug concentration causing 50% inhibition) values for each cell line were determined using “log (inhibitor) vs. normalized response – variable slope” equation. Combined treatment was performed as described for single drug assays with slight modifications: two drugs were simultaneously applied at defined concentrations (4x IC_50_, 2x IC_50_, 1x IC_50_, 0.5x IC_50,_ 0.25x IC_50_, 0.125x IC_50_), respectively.

For EGFR pathway studies, cellular proteins were extracted in RIPA lysis buffer containing phosphatase inhibitors and quantified using the Pierce^TM^ BCA protein assay (Thermo Scientific, MA, USA) 24h after treatment of SCaBER cells with single and combined application of cisplatin, gemcitabine and/or erlotinib. Drug concentrations were selected according to the CI-value-dependent synergisms/antagonisms. In parallel RNA was extracted using the Nucleospin RNA Plus Kit (Macherey-Nagel, 740984.50) according to the manufacturer's instructions.

**RNA interference of EGFR**

Cells were transfected with siTran 1.0 siRNA transfection reagent (Origene, Cat. No. TT300002) applying a siRNA directed against EGFR (Origene, Cat. No. SR320009A, 5’-UUCCUUUACGGUUUUCAGAAUAUCCAG-3’ (10 nM)), according to the manufacturer’s instructions. Cells were retreated with siRNA after 72 h to ensure sufficient EGFR knockdown. Commercial non-silencing control siRNA (nc siRNA) (Origene, Cat. No. SR30004, sense: 5’-CGUUAAUCGCGUAUAAUACGCGUAT-3’, antisense: 5’-AUACGCGUAUUAUACGCGAUUAACGAC-3’) served as negative control. Knockdown was verified by RT-PCR and western blot analysis after 48, 96 and 144 h. Functional analyses were performed every 24 h, starting 24 h after transfection. Cell viability was determined 72 h, 96 h, 120 h, 144 h and 168 h after transfection.

**Microarray analysis**

Total RNA was isolated from patient tumor material using the automated Maxwell 16 LEV simplyRNA Tissue Kit (Promega, AS1280) according to the manufacturer's instructions. RNA from primary cultured tumor cells and cell lines were was extracted using the Nucleospin RNA Plus Kit (Macherey-Nagel, 740984.50) according to the manufacturer's instructions. RNA quality was assessed with the RNA Screen Tape Assay using Agilent Tape Station 4200 (Agilent Technologies). For microarray analysis, each 300 ng RNA were transcribed and labeled with GeneChip wt PLUS Reagent Kit (Affymetrix) according to the manufacturer’s protocol. Processed samples were hybridized on Human Clariom D (Thermo Fisher Scientific, Waltham, MA, USA) gene chip and visualized with the Affymetrix staining kit. The Affymetrix Expression Console software was utilized for gene data analysis. With the help of the Transcriptome Analysis Console (TAC) 4.0 software (Applied Biosystem, Foster City, CA USA by Thermo Fisher Scientific), signals intensity was background adjusted, normalized, and log-transformed using the Signal Space Transformation (SST)-Robust Multi-Array Average algorithm (RMA).

**TCGA data acquisition**

Public datasets from the Cancer Genome Atlas (TCGA) network were used for *in silico* analysis of ERBB expression in muscle invasive bladder cancer (overall included n=408) [59]. Subtypes were classified and subsequently *EGFR*, *ERBB2*, *ERBB3* and *ERBB4* gene copy number variations (CNV; Affymetrix Genome-Wide SNP Array 6.0 with a fixed probe set and Illumina HiSeq Platforms; Level 3), somatic mutations (Illumina Genome Analyzer and Illumina HiSeq2000 DNASeq Platform; Level 3)), and mRNA expression were analyzed as previously described [22]. Quantile normalized mRNA expression values were log2-transformed to reduce skewness prior to any statistical tests.
