# Supplementary material for: EGFR activity addiction facilitates anti-ERBB based combination treatment of squamous bladder cancer
Source: Oncogene. 2020 Sep 25;39(44):6856–70. doi: 10.1038/s41388-020-01465-y (PMC7605436; doi:10.1038/s41388-020-01465-y)
Supplement: Supplementary file 2 — Supplementary Figure 1: ERBB receptor expression in urothelial, squamous bladder and head and neck cancer cell lines. [file 41388_2020_1465_MOESM2_ESM.docx]

**
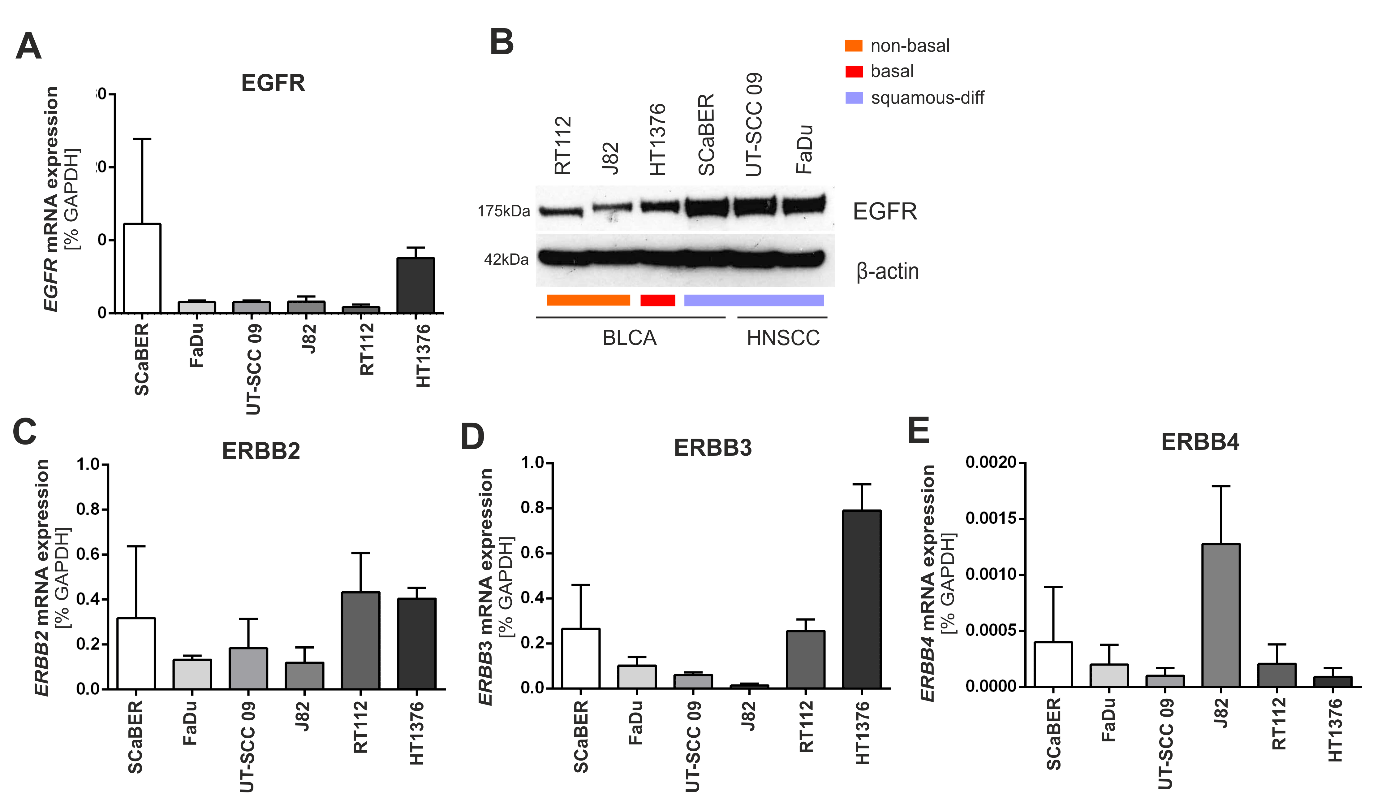
**

**Supplementary Figure 1: ERBB receptor expression in urothelial, squamous bladder and oropharyngeal cancer** **cell lines. (A)** Relative mRNA expression of EGFR normalized to corresponding GAPDH expression and visualized as relative expression in % of *GAPDH* expression. **(B)** Western blot shows EGFR protein expression. β-actin served as loading control**. (C-E)** mRNA expression of ERBB2 (B), ERBB3 (C) and ERBB4 (D) normalized to corresponding *GAPDH* expression and visualized as relative expression in % of *GAPDH* expression (SCaBER n=7, FaDu n=3, UT-SCC 09 n=3, J82 n=5, RT112 n=5, HT1376 n=3).
