# Supplementary material for: EGFR activity addiction facilitates anti-ERBB based combination treatment of squamous bladder cancer
Source: Oncogene. 2020 Sep 25;39(44):6856–70. doi: 10.1038/s41388-020-01465-y (PMC7605436; doi:10.1038/s41388-020-01465-y)
Supplement: Supplementary file 4 — Supplementary Figure 3: Densitometric evaluation of ERBB pathway activation and inhibition in J82 cells. [file 41388_2020_1465_MOESM4_ESM.docx]

**

**

**Supplementary Figure 3: Densitometric evaluation of ERBB pathway activation and inhibition in J82 cancer cells.** Densitometric analysis of detected protein levels is shown for EGFR, p-EGFR, ERK, p-ERK and p-AKT 24h after EGF stimulation and/or erlotinib treatment for J82. DMSO was used as untreated control and set to 100%. Total EGFR and ERK protein amount was normalized to loading controls (actin and/or tubulin) and DMSO control. Activated p-EGFR and p-ERK was then standardized to total EGFR and ERK, respectively.
