# Supplementary material for: EGFR activity addiction facilitates anti-ERBB based combination treatment of squamous bladder cancer
Source: Oncogene. 2020 Sep 25;39(44):6856–70. doi: 10.1038/s41388-020-01465-y (PMC7605436; doi:10.1038/s41388-020-01465-y)
Supplement: Supplementary file 5 — Supplementary Figure 4: ERBB signaling and receptor expression in HT1376 bladder cancer cells upon TKI treatment and EGF stimulation. [file 41388_2020_1465_MOESM5_ESM.docx]

**

**

**Supplementary Figure 4: ERBB signaling and receptor expression in HT1376 bladder cancer cells upon TKI treatment and EGF stimulation. (A)** Western blot analysis illustrates activation and inhibition of EGFR / p-EGFR (Tyr1068) and ERK / p-ERK (Thr202,Tyr204), 24h after EGF and/or erlotinib treatment. DMSO application was used as untreated control. β-actin (for EGFR) and tubulin (for ERK) served as loading control. **(B)** Relative mRNA expression of ERBB receptors (*EGFR, ERBB2, ERBB3* and *ERBB4*) normalized to corresponding DMSO control 24h after EGF and erlotinib treatment. *GAPDH* was used for standardization. FC: fold change. Vertical lines: + standard error of mean (SEM). **(C)** Densitometric analysis of detected protein levels is shown for EGFR, p-EGFR (Tyr1068), ERK, and p-ERK 24h after EGF stimulation and/or erlotinib treatment for J82. DMSO was used as untreated control and set to 100%. Total EGFR and ERK protein amount was normalized to loading controls (actin and/or tubulin) and DMSO control. Activated p-EGFR and p-ERK was then standardized to total EGFR and ERK, respectively.
