# Supplementary material for: EGFR activity addiction facilitates anti-ERBB based combination treatment of squamous bladder cancer
Source: Oncogene. 2020 Sep 25;39(44):6856–70. doi: 10.1038/s41388-020-01465-y (PMC7605436; doi:10.1038/s41388-020-01465-y)
Supplement: Supplementary file 6 — Supplementary Figure 5: ERBB receptor expression after siRNA mediated knockdown of EGFR in J82 cells. [file 41388_2020_1465_MOESM6_ESM.docx]

**
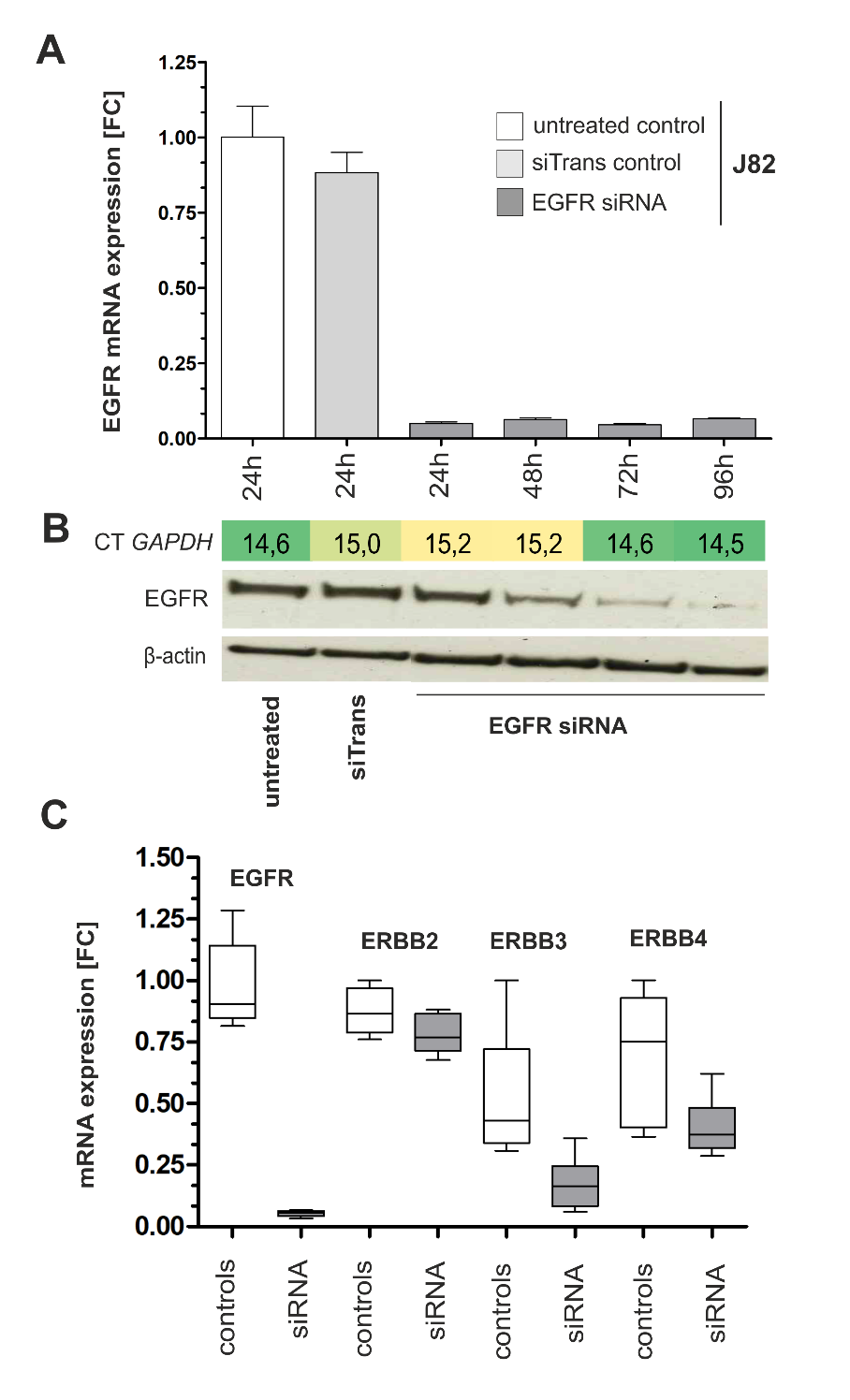
**

**Supplementary Figure 5: ERBB receptor expression after siRNA mediated knockdown of EGFR in J82 cells. (A)** siRNA mediated knockdown of EGFR in J82 cells. EGFR knockdown was observed on mRNA level after 24h (A) and on protein level after 48h **(B)** while no impact on both CT GAPDH and cell viability was observed**.** FC: fold change. Vertical lines: + SEM of triplicates. **(C)** Relative mRNA expression of ERBB receptors (*EGFR, ERBB2, ERBB3* and *ERBB4*) normalized to corresponding *GAPDH* expression is shown for siRNA controls and siRNA-mediated knockdown of EGFR (24-96h for each group).
