# Supplementary material for: EGFR activity addiction facilitates anti-ERBB based combination treatment of squamous bladder cancer
Source: Oncogene. 2020 Sep 25;39(44):6856–70. doi: 10.1038/s41388-020-01465-y (PMC7605436; doi:10.1038/s41388-020-01465-y)
Supplement: Supplementary file 7 — Supplementary Figure 6: ERBB receptor expression (EGFR, ERBB2, ERBB3) upon combined treatment in SCaBER cells [file 41388_2020_1465_MOESM7_ESM.docx]

**
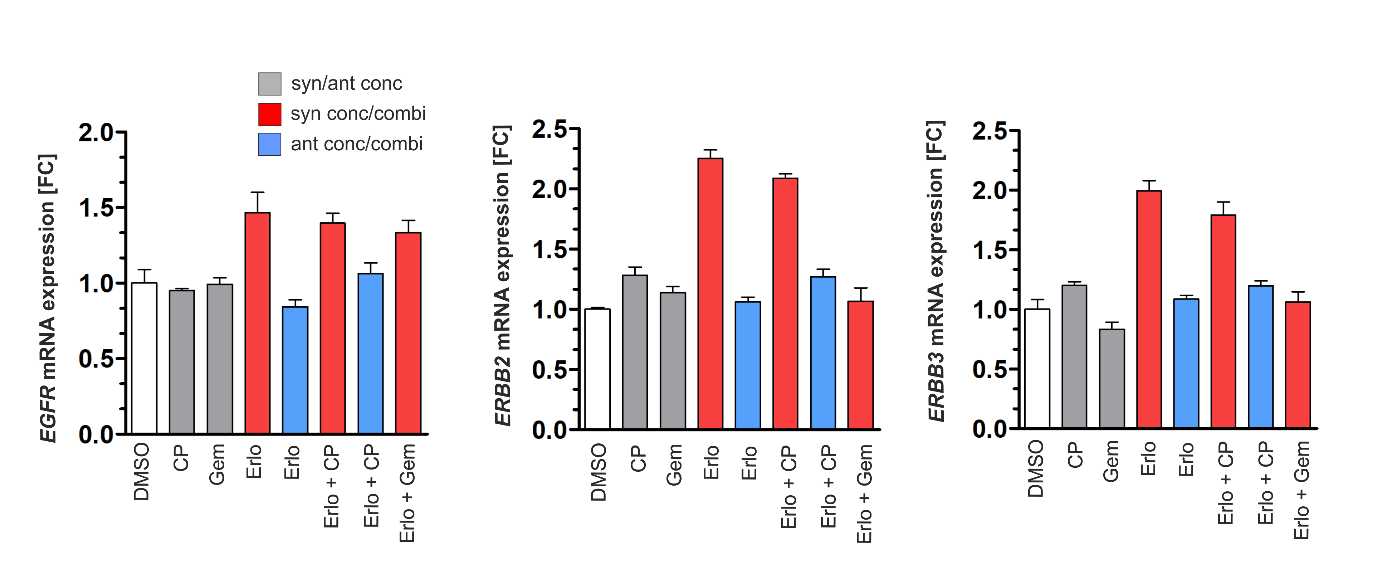
**

**Supplementary Figure 6:** **ERBB receptor expression (*EGFR, ERBB2, ERBB3*) upon combined treatment in SCaBER cells.** Relative mRNA expression of the ERBB receptors *EGFR, ERBB2* and *ERBB3* normalized to corresponding DMSO control 24h after treatment of SCaBER cells with indicated drugs. *GAPDH* was used for standardization. *ERBB4* mRNA expression is presented in Figure 5. Data were confirmed by n=3 independent replicates.
