# Supplementary material for: EGFR activity addiction facilitates anti-ERBB based combination treatment of squamous bladder cancer
Source: Oncogene. 2020 Sep 25;39(44):6856–70. doi: 10.1038/s41388-020-01465-y (PMC7605436; doi:10.1038/s41388-020-01465-y)
Supplement: Supplementary file 8 — Supplementary Figure 7: EGFR signaling in pSCC cells upon TKI treatment and EGF stimulation. [file 41388_2020_1465_MOESM8_ESM.docx]

**

**

**Supplementary Figure 7:** **EGFR signaling in pSCC cells upon TKI treatment and EGF stimulation.** Western blot analyses illustrate activation and inhibition of EGFR / p-EGFR (Tyr1068), ERK / p-ERK (Thr202,Tyr204), and p-AKT (Ser473) 24h after EGF and erlotinib treatment. DMSO application was used as untreated control. β-actin (for EGFR) and tubulin (for ERK) served as loading control. **(B)** Relative mRNA expression of ERBB ligands (*AREG, EREG* and *HB-EGF*) and the EGFR target gene *SOX9* normalized to corresponding DMSO control 24h after EGF and/or erlotinib treatment. *GAPDH* was used for standardization. DMSO application served as untreated control. FC: fold change. Vertical lines: + standard error of mean (SEM).
