# Supplementary material for: EGFR activity addiction facilitates anti-ERBB based combination treatment of squamous bladder cancer
Source: Oncogene. 2020 Sep 25;39(44):6856–70. doi: 10.1038/s41388-020-01465-y (PMC7605436; doi:10.1038/s41388-020-01465-y)
Supplement: Supplementary file 9 — Supplementary Table 1: Clinico-pathological data of urothelial, muscle-invasive bladder cancers without squamous characteristics used in this study. [file 41388_2020_1465_MOESM9_ESM.docx]

| **Supplementary Table 1: Clinico-pathological data of urothelial, muscle-invasive bladder cancers without squamous characteristics used in this study (MIBC, n=63)** | | | |
| --- | --- | --- | --- |
|  |  |  |  |
|  |  |  |  |
| **Variable** | | **n** | **%** |
|  |  |  |  |
| Patient age (years) | |  |  |
|  | median age = 74 |  |  |
|  | ≤74 | 32 | 50.8 |
|  | >74 | 31 | 49.2 |
| Gender | |  |  |
|  | Female | 21 | 33.3 |
|  | Male | 42 | 66.7 |
| Tumor stage | |  |  |
|  | pT1 | 3 | 4.8 |
|  | pT2 | 26 | 41.2 |
|  | pT3 | 23 | 36.5 |
|  | pT4 | 11 | 17.5 |
| Tumor grade | |  |  |
|  | G2 | 6 | 9.5 |
|  | G3 | 56 | 88.9 |
|  | na | 1 | 1.6 |
| Nodal status | |  |  |
|  | N0 | 25 | 39.7 |
|  | N1 | 9 | 14.3 |
|  | N2 | 1 | 1.6 |
|  | na | 28 | 44.4 |
| na=not available | | | |
