# Supplementary material for: EGFR activity addiction facilitates anti-ERBB based combination treatment of squamous bladder cancer
Source: Oncogene. 2020 Sep 25;39(44):6856–70. doi: 10.1038/s41388-020-01465-y (PMC7605436; doi:10.1038/s41388-020-01465-y)
Supplement: Supplementary file 10 — Supplementary Table 2: Detailed information on identified mutations in Sq-BLCA (SCC n=34, MIX n=40). [file 41388_2020_1465_MOESM10_ESM.docx]

| **Supplementary Table 2: Detailed information on identified mutations in Sq-BLCA (SCC n=34, MIX n=40)** | | | | | | |
| --- | --- | --- | --- | --- | --- | --- |
|  |  |  |  |  |  |  |
| **Type** | **TP53 (NM_000546)** | **FGFR3 (NM_001163213)** | **KRAS (NM_004985)** | **NRAS (NM_002524)** | **HRAS (NM_005343)** | **EGFR Mutation (NM_005228)** |
| SCC | c.586C>T, (p.Arg196*) | WT | WT | WT | WT | WT |
| SCC | c.892G>T, (p.Glu298*) | WT | WT | WT | WT | WT |
| SCC | c.778_779del, (p.Ser260Glnfs*3); c.536A>G, (p.His179Arg) | WT | WT | WT | WT | WT |
| SCC | c.524G>A, (p.Arg175His) | WT | na | na | na | WT |
| SCC | c.404G>T, (p.Cys135Phe) | WT | WT | WT | WT | WT |
| SCC | c.584T>C, (p.Ile195Thr) | WT | WT | WT | WT | WT |
| SCC | c.800G>C, (p.Arg267Pro) | WT | WT | WT | WT | WT |
| SCC | c.839G>C, (p.Arg280Thr) | WT | WT | WT | WT | WT |
| SCC | c.839G>C, (p.Arg280Thr) | WT | WT | WT | WT | WT |
| SCC | c.839G>C, (p.Arg280Thr) | WT | WT | WT | WT | WT |
| SCC | c.835G>C, (p.Glu285Gln) | WT | WT | WT | WT | WT |
| SCC | na | na | WT | WT | WT | WT |
| SCC | WT | c.746C>G, (p.Ser249Cys) | WT | WT | WT | WT |
| SCC | WT | c.746C>G, (p.Ser249Cys) | WT | WT | WT | WT |
| SCC | WT | c.746C>G, (p.Ser249Cys) | WT | WT | WT | WT |
| SCC | WT | c.746C>G, (p.Ser249Cys) | WT | WT | WT | WT |
| SCC | WT | WT | na | na | na | na |
| SCC | WT | WT | WT | WT | WT | WT |
| SCC | WT | WT | WT | WT | WT | WT |
| SCC | WT | WT | WT | WT | WT | WT |
| SCC | WT | WT | WT | WT | WT | WT |
| SCC | WT | WT | WT | WT | WT | WT |
| SCC | WT | WT | WT | WT | WT | WT |
| SCC | WT | WT | WT | WT | WT | WT |
| SCC | WT | WT | WT | WT | WT | WT |
| SCC | WT | WT | WT | WT | WT | WT |
| SCC | WT | WT | WT | WT | WT | WT |
| SCC | WT | WT | WT | WT | WT | WT |
| SCC | WT | WT | WT | WT | WT | WT |
| SCC | WT | WT | WT | WT | WT | WT |
| SCC | WT | WT | WT | WT | WT | WT |
| SCC | WT | WT | WT | WT | WT | WT |
| SCC | WT | WT | WT | WT | WT | WT |
| SCC | WT | WT | WT | WT | WT | WT |
| MIX | c.447_459del, (p.Thr150Alafs*16) | WT | WT | WT | WT | WT |
| MIX | c.460_473del, (p.Gly154Argfs*22) | WT | WT | WT | WT | WT |
| MIX | c.703del, (p.Asn235Thrfs*12) | WT | WT | WT | WT | WT |
| MIX | c.723del, (p.Cys242Alafs*5) | WT | WT | WT | WT | WT |
| MIX | c.853G>T, (p.Glu285*) | WT | WT | WT | WT | WT |
| MIX | c.991C>T, (p.Gln331*) | WT | WT | WT | WT | WT |
| MIX | c.413C>T, (p.Ala138Val) | c.746C>G, (p.Ser249Cys) | WT | WT | WT | WT |
| MIX | c.832C>T, (p.Pro278Ser) | WT | na | na | na | na |
| MIX | c.469G>T, (p.Val157Phe) | WT | WT | WT | WT | WT |
| MIX | c.530C>T, (p.Pro177Leu); c.659A>G, (p.Tyr220Cys) | WT | WT | WT | WT | WT |
| MIX | c.581T>G, (p.Leu194Arg) | WT | WT | WT | WT | WT |
| MIX | c.679T>C, (p.Ser227Pro); c.818G>A, (Arg273His) | WT | WT | WT | WT | WT |
| MIX | c.722C>G, (p.Ser241Cys) | WT | WT | WT | WT | WT |
| MIX | c.817C>T, (p.Arg273Cys) | WT | WT | WT | WT | WT |
| MIX | c.817C>A, (p.Arg273Ser) | WT | WT | WT | WT | WT |
| MIX | c.839G>A, (p.Arg280Lys) | WT | WT | WT | WT | WT |
| MIX | c.839G>C, (p.Arg280Thr) | WT | WT | WT | WT | WT |
| MIX | c.839G>C, (p.Arg280Thr) | WT | WT | WT | WT | WT |
| MIX | c..853G>A, (p.Glu285Lys) | WT | WT | WT | WT | WT |
| MIX | WT | c.746C>G, (p.Ser249Cys) | WT | WT | WT | WT |
| MIX | WT | c.746C>G, (p.Ser249Cys) | WT | WT | WT | WT |
| MIX | WT | WT | WT | WT | c.182A>T, (p.Gln61Leu) | WT |
| MIX | WT | WT | na | na | na | na |
| MIX | WT | WT | WT | WT | WT | WT |
| MIX | WT | WT | WT | WT | WT | WT |
| MIX | WT | WT | WT | WT | WT | WT |
| MIX | WT | WT | WT | WT | WT | WT |
| MIX | WT | WT | WT | WT | WT | WT |
| MIX | WT | WT | WT | WT | WT | WT |
| MIX | WT | WT | WT | WT | WT | WT |
| MIX | WT | WT | WT | WT | WT | WT |
| MIX | WT | WT | WT | WT | WT | WT |
| MIX | WT | WT | WT | WT | WT | WT |
| MIX | WT | WT | WT | WT | WT | WT |
| MIX | WT | WT | WT | WT | WT | WT |
| MIX | WT | WT | WT | WT | WT | WT |
| MIX | WT | WT | WT | WT | WT | WT |
| MIX | WT | WT | WT | WT | WT | WT |
| MIX | WT | WT | WT | WT | WT | WT |
| MIX | WT | WT | WT | WT | WT | WT |
| WT: wildtype; na: not available | | | | | | |
|  |  |  |  |  |  |  |
