# Supplementary material for: EGFR activity addiction facilitates anti-ERBB based combination treatment of squamous bladder cancer
Source: Oncogene. 2020 Sep 25;39(44):6856–70. doi: 10.1038/s41388-020-01465-y (PMC7605436; doi:10.1038/s41388-020-01465-y)
Supplement: Supplementary file 11 — Supplementary Table 3: Detailed information on amplification of EGFR and HER2/ERBB2 in Sq-BLCA [file 41388_2020_1465_MOESM11_ESM.docx]

| **Supplementary Table 3: Detailed information on amplification of EGFR and HER2/ERBB2 in Sq-BLCA** | | |
| --- | --- | --- |
|  |  |  |
| **Type** | **EGFR FISH (ratio)** | **HER2/ERBB2 FISH (ratio)** |
| Pure | 1.03 | 1.13 |
| Pure | 1.03 | 1.04 |
| Pure | na | na |
| Pure | 0.99 | 1.01 |
| Pure | 1.01 | 1.37 |
| Pure | 1.03 | 1 |
| Pure | na | na |
| Pure | 1.08 | 1.08 |
| Pure | Cluster | 0.96 |
| Pure | na | na |
| Pure | 1.01 | 0.85 |
| Pure | 1.05 | na |
| Pure | 1.01 | na |
| Pure | 1.06 | 1.2 |
| Pure | 1.06 | 0.98 |
| Pure | 1 | 1 |
| Pure | 0.98 | 0.53 |
| Pure | 1.07 | 0.92 |
| Pure | 0.98 | na |
| Pure | na | na |
| Pure | 0.98 | 0.7 |
| Pure | Cluster | 1.12 |
| Pure | na | na |
| Pure | 0.97 | 1.05 |
| Pure | 1 | 1.04 |
| Pure | 1.03 | 1.04 |
| Pure | 0.98 | na |
| Pure | 1.02 | 1.1 |
| Pure | 1.03 | 1.07 |
| Pure | 1.01 | 0.97 |
| Pure | 1 | 1.05 |
| Pure | 1.01 | 0.87 |
| Pure | 1.04 | 1.1 |
| Pure | 1.05 | 0.94 |
| Pure | Cluster | 0.62 |
| Pure | Cluster | 0.75 |
| Pure | na | 1.18 |
| Pure | 0.98 | 1.3 |
| Pure | 1.04 | 1.08 |
| Pure | 0.99 | 1.28 |
| Pure | 1.15 | 1.25 |
| Pure | 1.14 | 1.08 |
| Pure | 1.07 | 1.05 |
| Pure | 1.04 | 1.38 |
| Pure | 0.97 | 1.11 |
| Pure | 0.95 | na |
| Pure | 0.96 | na |
| Pure | 1.03 | 1.13 |
| Pure | 1.55 | 1.03 |
| Pure | 1.05 | 1.06 |
| Pure | 1 | 1.02 |
| Pure | 1.06 | 0.98 |
| Pure | 0.98 | 0.85 |
| Pure | 1 | 0.96 |
| Pure | 1.08 | 1.12 |
| Pure | 0.93 | 0.97 |
| Pure | 0.98 | 1.41 |
| Pure | 1.02 | 1.28 |
| Pure | 0.96 | 1.19 |
| Pure | 1.05 | 0.99 |
| Pure | 0.94 | 1.16 |
| Pure | 0.82 | 0.96 |
| Pure | 0.99 | 0.93 |
| Pure | 0.98 | 0.98 |
| Pure | 1.02 | 1.24 |
| Pure | 0.96 | 0.88 |
| Pure | Cluster | 0.96 |
| Pure | 1.29 | na |
| Pure | 1.03 | 1.01 |
| Pure | 1.06 | 1.16 |
| Pure | 0.99 | 1.05 |
| Pure | 0.96 | 1.02 |
| Pure | Cluster | 0.99 |
| Pure | 0.81 | 0.85 |
| Pure | 0.99 | 0.97 |
| Mix | 0.99 | 0.94 |
| Mix | Cluster | 1.03 |
| Mix | 1 | 0.95 |
| Mix | na | na |
| Mix | 1.07 | 0.94 |
| Mix | 0.45 | 1.01 |
| Mix | 0.97 | 1.11 |
| Mix | 1.03 | na |
| Mix | Cluster | 1.02 |
| Mix | 1.01 | 0.95 |
| Mix | 0.99 | 0.72 |
| Mix | 1.01 | 0.99 |
| Mix | 0.94 | na |
| Mix | 1.03 | 1.34 |
| Mix | 1.02 | 1.61 |
| Mix | 1.04 | na |
| Mix | na | na |
| Mix | 1.01 | 0.96 |
| Mix | 1.03 | 1.09 |
| Mix | na | 0.96 |
| Mix | 1.04 | 0.96 |
| Mix | 1.03 | 0.97 |
| Mix | 1.01 | 1.12 |
| Mix | 0.98 | 1.09 |
| Mix | 0.97 | 1.04 |
| Mix | 0.96 | 1.14 |
| Mix | 1.02 | 1.15 |
| Mix | 1.01 | 0.61 |
| Mix | 1.13 | 0.82 |
| Mix | 1.02 | 1.01 |
| Mix | 1.01 | na |
| Mix | 0.97 | 1.22 |
| Mix | 1.02 | 0.97 |
| Mix | 1.02 | 1.38 |
| Mix | na | na |
| Mix | 0.98 | na |
| Mix | 1.02 | 1.03 |
| Mix | 1.02 | 1.09 |
| Mix | 1.06 | 1.04 |
| Mix | 1.09 | 1.07 |
| Mix | 0.99 | 1.28 |
| Mix | 1.02 | 1.01 |
| Mix | 1.02 | 1.62 |
| Mix | 0.95 | 1.07 |
| Mix | 1.03 | 1.03 |
| Mix | 0.98 | 0.96 |
| Mix | 1.01 | 1.1 |
| Mix | 1 | 0.93 |
| Mix | 0.99 | 1.02 |
| Mix | Cluster | 0.91 |
| na: not available | | |
|  |  |  |
