# Supplementary material for: EGFR activity addiction facilitates anti-ERBB based combination treatment of squamous bladder cancer
Source: Oncogene. 2020 Sep 25;39(44):6856–70. doi: 10.1038/s41388-020-01465-y (PMC7605436; doi:10.1038/s41388-020-01465-y)
Supplement: Supplementary file 15 — Supplementary Table 7: PCR primer sequences for ERBB receptor, ligand and target gene expression analysis (intron spanning). [file 41388_2020_1465_MOESM15_ESM.docx]

| **Supplementary Table 7: PCR primer sequences for ERBB receptor, ligand and target gene expression analysis (intron spanning)** | | | |
| --- | --- | --- | --- |
|  |  |  |  |
| **Gene** | | **Primer sequence** | **Product size (Bp)** |
| EGFR | |  | 90 |
|  |  | 5’-GGGGCCGACAGCTATGAGAT-3’ |  |
|  |  | 5’-ACCTATTCCGTTACACACTTTGC-3’ |  |
| ERBB2 | |  | 106 |
|  |  | 5’-GCCGTGCTAGACAATGGAGA-3’ |  |
|  |  | 5’-TCAAGATCTCTGTGAGGCTTCG-3’ |  |
| ERBB3 | |  | 131 |
|  |  | 5’-ACATCGTGAGGGACCGAGAT-3’ |  |
|  | | 5’-GGTCTTGGTCAATGTCTGGCA-3’ |  |
| ERBB4 | |  | 104 |
|  | | 5’-ATGGCTTACAGGGGGCAAAC-3’ |  |
|  | | 5’-AGTGGGACCGTTACACCCTT-3’ |  |
| AREG | |  | 195 |
|  | | 5'-GAACGGTGTGGGGAAAAGTC-3' |  |
|  | | 5'-CACTGGAAAGAGGACCGACT-3' |  |
| EREG | |  | 180 |
|  | | 5'-CCGTCCACCAACCTTTAAGC-3' |  |
|  | | 5'-CTTGCGGCAACTCTGGATC-3' |  |
| HB-EGF | |  | 182 |
|  | | 5’-CACCAAACAAGGAGGAGCAC-3’ |  |
|  | | 5’-CCCATGACACCTCTCTCCAT-3’ |  |
| SOX9 | |  | 209 |
|  | | 5'-CGGAGGAAGTCGGTGAAGAA-3' |  |
|  | | 5'-CTGCACGTCGGTTTTGGG-3' |  |
| KRT6A | |  | 184 |
|  | | 5'-CTAAAGTGCGTCTGCTA-3' |  |
|  | | 5'-TGGGTGCTCAGATGGTATA-3' |  |
| KRT14 | |  | 111 |
|  | | 5'-GAACCTGGAGATTGAGCTGC-3' |  |
|  | | 5'-ATCTCCTGGATCTGGGCCA-3' |  |
| GAPDH | |  | 109 |
|  | | 5’-GAAGGTGAAGGTCGGAGTCA-3’ |  |
|  | | 5’-AATGAAGGGGTCATTGATGG-3’ |  |
