# Supplementary material for: EGFR activity addiction facilitates anti-ERBB based combination treatment of squamous bladder cancer
Source: Oncogene. 2020 Sep 25;39(44):6856–70. doi: 10.1038/s41388-020-01465-y (PMC7605436; doi:10.1038/s41388-020-01465-y)
Supplement: Supplementary file 13 [file 41388_2020_1465_MOESM12_ESM.docx]

Supplementary Table 4: Molecular characteristics of utilized cell lines

Cell line		Mutation	Expression	CNV
**SCaBER**				
	EGFR	WT	Over (3.32)	None
	ERBB2	WT	Normal	None
	ERBB3	WT	Normal	None
	ERBB4	WT	Normal	None
	KRAS	WT	Normal	None
	NRAS	WT	Normal	None
	HRAS	WT	Normal	None
**J82**				
	EGFR	WT	Normal	None
	ERBB2	R678Q	Normal	None
	ERBB3	WT	Normal	None
	ERBB4	R1045G	Normal	None
	KRAS	WT	Normal	None
	NRAS	WT	Normal	None
	HRAS	WT	Over (2.30)	None
**RT112**				
	EGFR	WT	Normal	None
	ERBB2	WT	Normal	None
	ERBB3	WT	Normal	None
	ERBB4	WT	Normal	None
	KRAS	WT	Normal	None
	NRAS	WT	Normal	None
	HRAS	WT	Normal	None
**HT1376**				
	EGFR	WT	Over (2.66)	None
	ERBB2	WT	Normal	None
	ERBB3	WT	Normal	None
	ERBB4	WT	Normal	None
	KRAS	WT	Under (-2.90)	Loss
	NRAS	WT	Normal	None
	HRAS	WT	Over (2.35)	None
**FaDu**				
	EGFR	WT	Over (2.66)	None
	ERBB2	WT	Normal	None
	ERBB3	WT	Normal	None
	ERBB4	WT	Normal	None
	NRAS	WT	Normal	None
	KRAS	WT	Normal	None
	HRAS	WT	Normal	None
**UT-SCC 09**				
	EGFR	WT	na	na
	ERBB2	na	na	na
	ERBB3	na	na	na
	ERBB4	na	na	na
	NRAS	WT	na	na
	KRAS	WT	na	na
	HRAS	WT	na	na

na=not available, all molecular cell line information was obtained through COSMIC (Cell Lines Project v88, released 19-MAR-19) and mutational status for EGFR, KRAS; NRAS and HRAS was additionally confirmed by Sanger sequencing. ERBB2 and ERBB4 mutations in J82 were confirmed by wohle exome sequencing (WES).
