# Supplementary material for: EGFR activity addiction facilitates anti-ERBB based combination treatment of squamous bladder cancer
Source: Oncogene. 2020 Sep 25;39(44):6856–70. doi: 10.1038/s41388-020-01465-y (PMC7605436; doi:10.1038/s41388-020-01465-y)
Supplement: Supplementary file 14 [file 41388_2020_1465_MOESM13_ESM.docx]

Supplementary Table 5: p-SCC associated gene signature

ID	Gene Symbol	Description
TC1200012597.hg.1	MGST1	microsomal glutathione S-transferase 1
TC1900011967.hg.1	PSG9	pregnancy specific beta-1-glycoprotein 9
TC1900010818.hg.1	PSG1	pregnancy specific beta-1-glycoprotein 1
TC0500012519.hg.1	SPARC	secreted protein, acidic, cysteine-rich (osteonectin)
TC1000007883.hg.1	SRGN	serglycin
TC1900010824.hg.1	PSG11	pregnancy specific beta-1-glycoprotein 11
TC1900010833.hg.1	PSG5	pregnancy specific beta-1-glycoprotein 5
TC1000006891.hg.1	VIM	vimentin
TC0X00007176.hg.1	SSX1	synovial sarcoma, X breakpoint 1
TC0800007899.hg.1	SULF1	sulfatase 1
TC1300008644.hg.1	POSTN	periostin, osteoblast specific factor
TC1900011963.hg.1	PSG6	pregnancy specific beta-1-glycoprotein 6
TC1900011965.hg.1	PSG2	pregnancy specific beta-1-glycoprotein 2
TC1400009214.hg.1	BMP4	bone morphogenetic protein 4
TC0X00007298.hg.1	SSX2B	synovial sarcoma, X breakpoint 2B
TC0X00011368.hg.1	SSX2; SSX2B	synovial sarcoma, X breakpoint 2; synovial sarcoma, X breakpoint 2B
TC0X00011413.hg.1	L1CAM	L1 cell adhesion molecule
TC1000011923.hg.1	GFRA1	GDNF family receptor alpha 1
TC1900011960.hg.1	PSG3	pregnancy specific beta-1-glycoprotein 3
TC0400011192.hg.1	PLAC8	placenta specific 8
TC1400010772.hg.1	DIO2	deiodinase, iodothyronine, type II
TC0100015830.hg.1	FLG	filaggrin
TC0900012219.hg.1	CDKN2A	cyclin-dependent kinase inhibitor 2A
TC0100007206.hg.1	CDA	cytidine deaminase
TC0200011093.hg.1	ALPP	alkaline phosphatase, placental
TC1600010732.hg.1	NQO1	NAD(P)H dehydrogenase, quinone 1
TC1200009892.hg.1	OLR1	oxidized low density lipoprotein (lectin-like) receptor 1
TC0700012684.hg.1	AKR1B1	aldo-keto reductase family 1, member B1 (aldose reductase)
TC1900011961.hg.1	PSG8	pregnancy specific beta-1-glycoprotein 8
TC1000008628.hg.1	ABCC2	ATP binding cassette subfamily C member 2
TC0500008125.hg.1	RHOBTB3	Rho-related BTB domain containing 3
TC1000011400.hg.1	ANKRD1	ankyrin repeat domain 1 (cardiac muscle)
TC1000007641.hg.1	DKK1	dickkopf WNT signaling pathway inhibitor 1
TC2000006444.hg.1	TRIB3	tribbles pseudokinase 3
TC2100007446.hg.1	COL6A1	collagen, type VI, alpha 1
TC0200010239.hg.1	COL3A1; MIR3606	collagen, type III, alpha 1; microRNA 3606
TC0700008358.hg.1	COL1A2	collagen, type I, alpha 2
TC1100012535.hg.1	PVRL1	poliovirus receptor-related 1 (herpesvirus entry mediator C)
TC1700012202.hg.1	KRT17P2	keratin 17 pseudogene 2
TC0100007486.hg.1	SFN	stratifin
TC1500010634.hg.1	CERS3	ceramide synthase 3
TC0100010035.hg.1	S100A7A	S100 calcium binding protein A7A
TC2000008381.hg.1	JAG1	jagged 1
TC0200006831.hg.1	VSNL1	visinin like 1
TC1400010777.hg.1	TC2N	tandem C2 domains, nuclear
TC0500012107.hg.1	CXCL14	chemokine (C-X-C motif) ligand 14
TC2100007451.hg.1	COL6A2	collagen, type VI, alpha 2
TC0200016193.hg.1	COL6A3	collagen, type VI, alpha 3
TC2000009317.hg.1	SULF2	sulfatase 2
TC1200010756.hg.1	KRT6B	keratin 6B, type II
TC0100006931.hg.1	PDPN	podoplanin
TC0400012173.hg.1	SFRP2	secreted frizzled-related protein 2
TC0100010020.hg.1	SPRR1B	small proline-rich protein 1B
TC1100012474.hg.1	MPZL2	myelin protein zero-like 2
TC0X00008785.hg.1	BGN	biglycan
TC1800009268.hg.1	DSC2	desmocollin 2
TC1900007205.hg.1	CASP14	caspase 14
TC1200010757.hg.1	KRT6C	keratin 6C, type II
TC1300008250.hg.1	GJB6	gap junction protein beta 6
TC1300008249.hg.1	GJB2	gap junction protein beta 2
TC0100010018.hg.1	SPRR3	small proline-rich protein 3
TC1800007010.hg.1	DSG1	desmoglein 1
TC1700007124.hg.1	KRT16P1	keratin 16 pseudogene 1
TC0100015862.hg.1	S100A7	S100 calcium binding protein A7
TC0100015847.hg.1	SPRR2D	small proline-rich protein 2D
TC0400007344.hg.1	UCHL1	ubiquitin C-terminal hydrolase L1
TC0100017844.hg.1	NID1	nidogen 1
TC1700010677.hg.1	KRT19	keratin 19, type I
TC0100010017.hg.1	SPRR1A	small proline-rich protein 1A
TC1700008920.hg.1	ITGB4	integrin beta 4
TC0700006629.hg.1	FSCN1	fascin actin-bundling protein 1
TC2000007479.hg.1	PI3	peptidase inhibitor 3, skin-derived
TC1700012203.hg.1	KRT16P4	keratin 16 pseudogene 4
TC0X00009611.hg.1	SLC38A5	solute carrier family 38, member 5
TC2000009216.hg.1	SLPI	secretory leukocyte peptidase inhibitor
TC1800009308.hg.1	SERPINB3	serpin peptidase inhibitor, clade B (ovalbumin), member 3
TC0100008921.hg.1	CLCA2	chloride channel accessory 2
TC0600013998.hg.1	THBS2	thrombospondin 2
TC1200006555.hg.1	CCND2	cyclin D2
TC0100018492.hg.1	SPRR2A	small proline-rich protein 2A
TC0300009843.hg.1	TP63	tumor protein p63
TC0900008847.hg.1	LCN2	lipocalin 2
TC0100010032.hg.1	S100A9	S100 calcium binding protein A9
TC0200010980.hg.1	CCL20	chemokine (C-C motif) ligand 20
TC0100015858.hg.1	S100A8	S100 calcium binding protein A8
TC1400008705.hg.1	SLC7A8	solute carrier family 7 (amino acid transporter light chain, L system), member 8
TC1000012121.hg.1	OAT	ornithine aminotransferase
TC1200012601.hg.1	SLCO1B3	solute carrier organic anion transporter family, member 1B3
TC1800007013.hg.1	DSG3	desmoglein 3
TC1700010676.hg.1	KRT15; MIR6510	keratin 15, type I; microRNA 6510
TC0100015155.hg.1	VAV3	vav 3 guanine nucleotide exchange factor
TC0600006869.hg.1	DSP	desmoplakin
TC1700009911.hg.1	KRT17P1	keratin 17 pseudogene 1
TC1800007543.hg.1	SERPINB5	serpin peptidase inhibitor, clade B (ovalbumin), member 5
TC1200010758.hg.1	KRT6A	keratin 6A, type II
TC0100016018.hg.1	CRABP2	cellular retinoic acid binding protein 2
TC0500010553.hg.1	FYB	FYN binding protein
TC1700010682.hg.1	KRT17	keratin 17, type I
TC0200007506.hg.1	EPCAM	epithelial cell adhesion molecule
TC0100011446.hg.1	MIR205HG; MIR205	MIR205 host gene; microRNA 205
TC1800009307.hg.1	SERPINB4	serpin peptidase inhibitor, clade B (ovalbumin), member 4
TC1100012131.hg.1	MMP1	matrix metallopeptidase 1
TC1700010680.hg.1	KRT14	keratin 14, type I
TC0600007377.hg.1	HIST1H2BM	histone cluster 1, H2bm
TC1800007547.hg.1	SERPINB13	serpin peptidase inhibitor, clade B (ovalbumin), member 13
[illegible]	ESRP1	epithelial splicing regulatory protein 1
