# Supplementary material for: EGFR activity addiction facilitates anti-ERBB based combination treatment of squamous bladder cancer
Source: Oncogene. 2020 Sep 25;39(44):6856–70. doi: 10.1038/s41388-020-01465-y (PMC7605436; doi:10.1038/s41388-020-01465-y)
Supplement: Supplementary file 15 [file 41388_2020_1465_MOESM14_ESM.docx]

Supplementary Table 6: Primer sequences for Sanger sequencing of FFPE Material

Gene		Primer sequence	Annealing Temperature	Product size (bp)
EGFR				
	Exon 18	5’-CTGAGGTGACCCTTGTCTCTGTGTTCTT-3’<br>5’-AGAGGCCTGTGCCAGGGACCTTA-3’	60°C	186
	Exon 19	5’-GCATGTGGCACCATCTCACAA-3’<br>5’-CCTGAGGTTCAGAGCCATGGA-3’	60°C	204
	Exon 20	5’-CCATGCGAAGCCACACTGA-3’<br>5’-CGTATCTCCCTTCCCTGATTACC-3’	60°C	248
	Exon 21	5’-GCAGAGCTTCTTCCCATGATGA-3’<br>5’-GCTGACCTAAAGCCACCTCCT-3’	60°C	236
KRAS				
	Exon 2	5’-AGGCCTGCTGAAAATGACTG-3’<br>5’-GGTCCTGCACCAGTAATATGCA-3’	65°C	180
	Exon 3	5’-CACTGTAATAATCCAGACTGTG-3’<br>5’-AATTACTCCTTATGTCAGCTT-3’	56°C	259
	Exon 4	5’-TTAAAAGAGTTAAGGACTCTGA-3’<br>5’-GCAGAAAACAGATCTGTATTTA-3’	56°C	190
NRAS				
	Exon 2	5’-GATGTGGCTCGCCAATTAAC-3’<br>5’-CACTGGGCCTCACCTCTATG-3’	65°C	183
	Exon 3	5’-CAAGTGGTTATAGATGGTGAAACC-3’<br>5’-AAGATCATCCTTTCAGAGAAAATAAT-3’	56°C	201
	Exon 4	5’-AGATTAAGCGAGTAAAAGACTCGG-3’<br>5’-TGCAAACTCTTGCACAAATGCTG-3’	60°C	193
HRAS				
	Exon 2	5’-CAGGAGACCCTGTAGGAGGA-3’<br>5’-TCGTCCACAAAATGGTTCTG-3’	55°C	139
	Exon 3	5’-GTGGTCATTGATGGGGAGAC-3’<br>5’-TGGTGTTGTTGATGGCAAAC-3’	55°C	133
	Exon 4	5’-CTCAGGGAGCAGATCAAACG-3’<br>5’-CTAGCTGTGGGGTGGAGAG-3’	55°C	193
FGFR3				
	Exon 7	5’-AGTGGCGGTGGTGGTGAGGGAG-3’<br>5’-TGTGCGTCACTGTACACCTT-3’	63°C	161
	Exon 10	5’-GCCAGGCCAGGCCTCAAC-3’<br>5’-CTTGAGCGGGAAGCGGGAGATCTTG-3’	65°C	219
	Exon 15	5’-TGGTGACCGAGGACAACGTGATG-3’<br>5’-CTCTGGTGAGTGTAGACTCG-3’	63°C	237
TP53				
	Exon 5	5’-TGCCGT CTTCCAGTTGCTTTATC-3’<br>5’-GCAATCAGTGAGGAATCAGAGGC-3‘	60°C	332
	Exon 6	5’-AGCAGCTGGGGCTGGAGAG-3’<br>5’-CTGGAGGCCCACTGACAAC-3’	63°C	254
	Exon 7	5’-CCAAGGCGCACTGGCCTCA-3’<br>5’-AGAGGCAAGCAGAGGCTGG-3’	63°C	214
	Exon 8	5’-CTGATTTCCTTACTGCCTC-3’<br>5’-CTGCACCCTTGGTCTCCTC-3’	60°C	228
	Exon 9	5’-GTTATGCCTCAGATTCACTT-3’<br>5’-CGGCATTTTGAGTGTTAGAC-3’	55°C	183
